# Immune regulatory functions of biologically active proteins from edible fungi

**DOI:** 10.3389/fimmu.2022.1034545

**Published:** 2023-01-12

**Authors:** Juan Xu, Dazhong Xu, Qiuhui Hu, Ning Ma, Fei Pei, Anxiang Su, Gaoxing Ma

**Affiliations:** College of Food Science and Engineering, Nanjing University of Finance and Economics, Collaborative Innovation Center for Modern Grain Circulation and Safety, Nanjing, China

**Keywords:** edible mushrooms, medicinal fungus, proteins, macrophages, immune regulation, cytokines

## Abstract

Proteins from edible mushrooms have a variety of biological activities. Here, thirteen precious edible mushrooms such as *Ophiocordyceps sinensis*, *Ganoderma lucidum*, and *Morchella esculenta* and nine common edible mushrooms such as *Flammulina velutipes*, *Pleurotus ostreatus*, and *Pleurotus eryngii*, etc., from which their proteins were extracted, their composition analyzed and their immunomodulatory activity assessed. Rare mushrooms are a species of edible mushrooms with higher edible value and medicinal value than common edible mushrooms. The results showed that all the different edible mushroom crude proteins increased the proliferation and phagocytosis of mouse macrophages, and we found that these edible mushroom proteins affected the secretion of reactive oxygen species and nitric oxide by mouse macrophages. Further studies on cytokines secreted by mouse macrophages showed a significant increase in pro-inflammatory cytokines, suggesting that edible mushroom proteins promote the polarisation of macrophages into classical M1-type macrophages, further demonstrating that edible mushroom proteins enhance immunity. It was also found that the immunomodulatory activity of the precious edible mushroom proteins was significantly higher than that of the common edible mushroom proteins. These results have important implications for the processing and product development of edible mushroom proteins.

## Introduction

The immunomodulatory effects of mushroom compounds have been demonstrated in many studies during the last decade (1–7). Active pharmaceutical chemicals derived from any biological source were shown to be the safest and most innocuous throughout the investigation of prospective substitutes. Due to their great nutritional value and variety of bioactive components, mushrooms are one of those that the food and biopharmaceutical sectors are always interested in using (8). The dry matter of mushrooms contains about 10-40% protein, 2-8% fat, 3-28% carbohydrate, and 3-32% fiber. In addition to this, many biologically active mushroom metabolites, such as lectins, terpenoids, peptides, and glycoproteins, play an important role in regulating and enhancing the composition of the immune system (9). Several scientific studies and clinical applications have shown that mushrooms can act as anticancer, anti-inflammatory, antibacterial, anti-allergic, and anti-diabetic immunomodulators because they are rich in bioactive metabolites and other phytochemicals (10–12). Due to their great medicinal and nutritional value, the FDA considers mushrooms a suitable health food (13).

For many years, proteins, polysaccharides, lipids, and phenolics derived through mushrooms are of great interest for their immunomodulatory and anticancer properties. In modern medical research and the pharmaceutical industry, immunomodulators are the main focus agents (14). Several mushroom-derived polysaccharides have been isolated from modulating the innate and adaptive human immune system and from protecting against autoimmune diseases through cytokine production interacting with immune cells such as NK cells, macrophages, lymphocytes, and dendritic cells (15–20). For example, polysaccharides such as shiitake mushroom polysaccharides, polysaccharide peptides, cleaved polysaccharides, and polysaccharide K (Kerstin) are used in cancer immunotherapy (21). Several scientific studies have shown that glucan, mannan, galactan, and fructan are some other well-studied polysulfated mucopolysaccharides with potential immunostimulatory activity (22). Fat is a small part of mushrooms that may play a role in regulating lipid levels. In fact, polyunsaturated fatty acids can lower serum cholesterol (23). Ergosterol and tocopherols have antioxidant properties that prevent cancer, cardiovascular diseases, and degenerative diseases. Linoleic acid reduces cardiovascular disease, triglyceride levels, and blood pressure (8) and also has anti-inflammatory activity (3). The lipids contained in mushrooms have anti-inflammatory properties because of their high content of unsaturated fatty acids, and mouse macrophages treated with Badia imbricata biomass extract showed anti-inflammatory effects (24).

Nowadays, polysaccharides were the first active ingredients in medicinal fungi, and most of the studies on active substances in medicinal fungi focused on polysaccharides, which were therefore considered to be the most important active factors in medicinal fungi (2), but proteins as the main immune function components have not been studied much, and there are mainly no studies related to the comparison of immune active proteins in different mushrooms. With the development of research on various bioactive proteins of medicinal fungi, this view has been gradually changed (3–8), especially the discovery of fungal immunomodulatory proteins has provided new ideas to study the pharmacological mechanisms of medicinal fungi. Many studies have investigated several bioactive proteins from different mushrooms with potent antitumor, and immunostimulatory functions from *Flammulina velutipes*, *Volvariella volvacea*, *G. tsugae*, *Auricularia polytricha*, *Pleurotus citrinophora*, *Poria cocos*, *Ganoderma sinens*, *Trametes versicolor*, *Chroogomphis rutilus*, *Ganoderma atrum*, *Stachybotrys chlorohalonata*, and *Dichomitus squalens* were isolated from five additional immunomodulatory proteins (1, 3, 6, 25–33), The names are FIP- fve, FIP-vvo, FIP-gts, APP, PCiP, TVP, etc. These proteins have immunomodulatory, anticancer, and antiviral activities and are non-toxic. Thus, fungal immunomodulatory proteins are a class of proteins with great potential to be developed as immune pharmaceuticals for the treatment of tumors and immune system diseases. However, most of them have studied the immunomodulatory effects of one or a few edible mushrooms individually, and so far, no systematic studies have been conducted on the strength of immunomodulatory effects of common and precious mushrooms. In the present study, we will discuss systematically for the first time the strength of immunomodulatory activities of twenty-two mushroom proteins, namely *Ordyceps militaris*, *Coriolus versicolor*, *Inonotus obliquus*, *Termitornyces albuminosus*, *Morchella esculenta*, *Ophiocordyceps sinensis*, *Boletus*, *Russula alutacea*, *Ganoderma lucidum*, *Grifola frondosa*, *Tuber melanosporum*, *Phellinus igniarius*, T*richoloma matsutake Singer*, *Volvariella volvacea*, *Pleurotus eryngii*, *Pleurotus ostreatus*, *Agaricus bisporus*, *Flammulina velutipes*, *Hericium erinaceus*, *Agrocybe aegerita*, *Lentinula edodes*, *Hypsizygus marmoreus*.

## Materials and methods

### Materials and general methods

*C. militaris* was purchased from Xinbin Manchu Autonomous County, Fushun City, Liaoning Province; *C. versicolor* was purchased from Changbai Mountain Specialty Products Company, Jilin Province; *I. obliquus* was purchased from Jilin Xiang Xiaobei Biotechnology Co. Ltd.; *M. esculenta* from Kunming Zeyu Trade Co.; *O. sinensis* from Naju Farmers’ Market, Tibet; *R. alutacea* from Kunming Zeyu Trade Co. Ltd.; *Boletus*, *G. frondosa*, *T. melanosporum* from Kunming Kangjiale Biotechnology Co.; *T. matsutake (S. Ito & S. Imai) Singer* was purchased from the local market in Linzhi, Tibet; *P. eryngii*, *V. volvacea*, *A. aegerita* were purchased from Fujian Shenger Food Co. Ltd.; *A. bisporus*, *F. velutipes*, *L. edodes* purchased from Yunnan Lakeview E-commerce Co. PBS, sodium tetraborate decahydrate lignin content assay kit, cellulose content assay kit, all purchased from Solebro; LPS, bca kit, cck-8 kit, neutral red kit, nitric oxide kit, ROS kit, Mouse Interleukin-1β Enzyme-Linked ImmunoSorbent Assay Kit, Mouse Interleukin-6 Enzyme-Linked ImmunoSorbent Assay Kit, Mouse Tumor Necrosis Factor-α Enzyme-Linked ImmunoSorbent Assay Kit, Mouse Interferon-γ Enzyme-Linked ImmunoSorbent Assay Kit, penicillin-streptomycin solution, were purchased from Biyuntian; DMEM high sugar medium and FBS (from Australia) were purchased from Gibco; galacturonic acid was purchased from Beijing Baidi Biological Company; m-hydroxybiphenyl was purchased from Tokyo Kasei Kogyo Co.

### Determination of protein content of edible mushrooms

Determination of protein content of twenty-two edible mushrooms according to the previous improved method (34).

### Preparation of proteins from twenty-two kinds of edible mushroom

The preparation method of different proteins was based on the previous work and improved. The method of protein extraction was improved based on previous studies in the laboratory (35). Firstly, twenty-two edible mushroom seed entities were dried to constant weight at 50°C, crushed in a pulverizer, and passed through a 100 mesh sieve. Ultra-pure water was added at a ratio of 1:25 (g/mL). The extract was repeatedly freeze-thawed at 20°C 3 times. The pH value was adjusted to 12.0 with 1 mol/L NaOH solution, and the extracts were extracted in a water bath at 40 °C for 180 min. The supernatant was extracted by centrifugation and titrated to isoelectric point 4.5 with 1 mol/L HCl, and the supernatant was discarded by centrifugation to obtain *C. militaris* extract.

Ultra-pure water was added at 1:27.5, 1:15, 1:15,1:25,1:20 and 1:15 (g/mL) feed-to-liquid ratios, respectively, and the pH values were adjusted to 11, 10, 11,10.5, 11.4 and 10 with 1 mol/L NaOH solution, respectively, in water baths at 40.5 °C, 57 °C, 40 °C, 35 °C,42 °C and 57°C for 180 min, 20 min, 25 min, 150 min, 240 min and 20 min, respectively. The extract was sonicated for 20 min, 20 min, 90 min, 30 min,60 min and 30 mim at a power of 400 W and a temperature of 30°C, respectively. The supernatant was centrifuged and titrated with 1 mol/L HCl to isoelectric points of 3.6, 3.6, 3.6, 3.5,3.0 and 3.6 and then the supernatant was discarded by centrifugation to obtain *R. alutacea* extract, *F. velutipes* extract, *T. albuminosus* extract, T*. matsutake Singer* extract (36), *C. versicolor* extract and *P. eryngii* extract (37). Ultra-pure water was added at 1:50, 1:40, 1:120, 1:25, 1:20, 1:25, and 1:40 (g/mL) solid-liquid ratios, respectively.The pH values were adjusted to 12.0, 8.0, 10.0, 10.4, 11.0, 10.0, 13.0, respectively, with 1 mol/L NaOH solution.Heating in a water bath at 60°C, 30°C, 65°C, 65°C, 55°C, 40°C, and 40°C for 60 min, 180 min, 60 min, 60 min, 120 min, 30 min, 180 min, respectively. The supernatant was then centrifuged for 20 min, 20 min, 21.8 min, 60 min, 30 min, and 20 min at a power of 400 W, 120 W, 120 W, 758.6 W, 450 W, 300 W, 500 W, and 30°C, respectively, and the supernatant was extracted with ultrasonic-assisted extraction. The supernatant was then centrifuged and titrated with 1 mol/L HCl to the isoelectric points of 4.2, 4.0, 4.0, 3.05, 3.5, 4.0, 3.5, and then the supernatant was discarded. Hericium erinaceus extract, Tuber melanosporum extract, Volvariella volvacea extract, and Grifola frondosa extract.Ultra-pure water was added at 1:20, 1:30, 1:30, 1:50, 1:45, 1:50, 1:30, 1:25 (g/mL) solid-liquid ratios, respectively.The pH values were adjusted to 11.0, 12.0, 12.0, 12.0, 10.0, 11.8, 11.0, 12.0, respectively, with 1 mol/L NaOH solution.Heating in a water bath at 30°C, 50°C, 35°C, 60°C, 50°C, 55°C, 40°C, and 40°C for 30 min, 90 min, 180 min, 60 min, 180 min, 90 min, 60 min, and 60 min, respectively. The supernatant was then centrifuged for 40 min, 60 min, 120 min, 60 min, 30 min, 90 min, 30 min, and 40 min at a power of 400 W, 500 W, 400 W, 400 W, 350 W, 600 W, 500 W, and 30 °C, respectively. The supernatant was then centrifuged and titrated with 1 mol/L HCl to the isoelectric points of 3.4, 4.0, 3.0, 3.4, 4.0, 3.4, 3.2, 4.0, and then the supernatant was discarded. *I. obliquus* extract, *P. igniarius* extract, *O. sinensis* extract, *M. esculenta* extract, *L. edodes* extract, *H. marmoreus* extract, *P. ostreatus* extract, *A. bisporus* extract were obtained.

The extracts were washed 5 times with distilled water, dissolved in a small amount of water, pH adjusted to neutral, and desalted by dialysis in a dialysis bag with distilled water for 72 hours. During this period, the dialysate was changed several times and freeze-dried under vacuum for 72 h. Twenty-two edible bacteria *O. sinensis* protein (OSP), *C. militaris* protein (CMP), *A. aegerita* protein (AAP), *C. versicolor* protein (CVP), *P. igniarius* protein (PIP), *G. lucidum* protein (GLP), *G. frondosa* protein (GFP), *M. esculenta* protein (MEP), I. *I. obliquus* protein (IOP), *Boletus* protein (BP), T. matsutake (S. Ito & S. Imai) Singer protein (TMSP), *R. alutacea* protein (RAP), *T. albuminosus* protein (TAP), *T. melanus* protein (TAP), and *G. lucidum* protein (GLP). TAP), *T. melanosporum* protein (TMP), *P. eryngii* protein (PEP), *F. velutipes* protein (FVP), *V. volvacea* protein (VVP), *H. erinaceus* protein (HEP), *P. ostreatus* protein (POP), *L. edodes* protein (LEP), *A. bisporus* protein (ABP), *H. marmoreus* protein (HMP).

### Determination of crude protein composition of edible mushrooms

The protein content of crude protein was determined by bca kit with bovine serum protein as standard; the polysaccharide content of the crude extract was determined by phenol-sulfuric acid method with glucose as standard (38); the cellulose content of the crude extract was determined by cellulose kit; the lignin content was determined by lignin content test kit; the ash content of the crude extract was determined by direct ash method; the moisture content of crude protein was determined by thermal drying method; the glyoxylate content of the crude extracts was determined by the m-hydroxyphenyl method.

### Cell culture

Macrophage RAW 264.7 was obtained from Wuhan Pronosai Life Sciences Co. Primary 264.7 macrophages were preserved in high sugar medium containing 10% fetal bovine serum. All media were supplemented with 100 mg/ml penicillin-streptomycin solution (35)

### Effect of active proteins of edible bacteria on RAW 264.7 viability

Macrophages were recovered and inoculated in 96-well cell culture plates (2000 cells/per well) with 200 μL of macrophage culture medium per well, and the culture medium was changed every day. The 96-well plates were incubated at 37°C in a cell culture incubator containing 5% CO2 until the logarithmic growth phase. Macrophages at the logarithmic growth stage were incubated with different concentrations of edible protein solution (25, 50, 100, 200 μg/mL), and a blank control was set up for 24 h. 20 μL of cck-8 solution was added to each well and incubated in the cell incubator for 1 h. The absorbance at λ=450 nm was measured by enzyme marker. Macrophages cultured in normal 96-well plates were used as blank controls for the same treatment. The toxicity and multiplication rate of macrophages was calculated as follows.


$$Proliferation\;rates=\frac{OD_{sample}}{OD_{control}}x100\%\text{ Figures}$$

Where _ODcontrol indicates the_ absorbance of cells cultured in blank wells and _ODsample_ indicates the absorbance of cells cultured in sample wells.

### Effect of active proteins of edible bacteria on the phagocytosis of neutral red by RAW 264.7

Macrophages inoculated in 96 wells were spiked and incubated for 24 h. 20 μL of neutral red solvent was added to each well and incubated for 20 min, and 100 μL of cell lysate was added. The absorbance at λ=570 nm was measured by an enzyme marker, where λ=690 nm was used as the reference wavelength. Macrophages cultured in normal 96-well plates were treated identically as a blank control group. Among them, the group with added lipopolysaccharide was made as a positive control.

### Effect of active proteins of edible bacteria on ROS secretion by RAW 264.7

Cells at the logarithmic growth stage were added to a 96-well cell culture (black plate) at a density of 1 × 10^6^ cells/mL, 100 μL per well, and incubated at 37°C in a 5% CO2 incubator for 24 h. After the supernatant was carefully discarded, 100 μL of DMEM basal medium, different concentrations (50 ~ 200 μg/mL) of edible bacterial protein solution, LPS (1 μ g/mL, prepared with DMEM basal medium), and three replicate wells were set up for each group. After 24 h of incubation, the supernatant was carefully discarded, and 100 μL of DCFH-DA solution (10 μmol/L) was added to each well, and the incubation was continued at 37°C for 20 min. The fluorescence intensity of each well was measured by an enzyme marker with an excitation wavelength of 488 nm and an emission wavelength of 525 nm. Among them, the group with added lipopolysaccharide was used as a positive control.

### Effect of active proteins of edible bacteria on the release of nitric oxide from RAW 264.7

Macrophages inoculated in 96-well plates were added to the samples and incubated for 24 h. The supernatant was aspirated. To the supernatant, 100 μL of Griess reagents solvent was added and incubated for 10 min at 25°C. The absorbance at λ=540 nm was measured by enzyme marker. The absorbance at the corresponding concentration was obtained by diluting 1 mol/L sodium nitrite solution to a concentration gradient solution of 0, 1, 2, 5, 10, 20, 40, 60, and 100 μmol/L using the cell culture solution and doing the same treatment as the experimental group. With the concentration as the horizontal coordinate and the corresponding absorbance as the vertical coordinate, a linear regression standard curve was plotted to obtain the corresponding calculation formula. Based on the absorbance obtained from the experimental group, the amount of nitric oxide released from macrophages was calculated. Among them, the group with the addition of lipopolysaccharide was used as a positive control (39).

### Comparison of the immune activity of twenty-two edible mushroom proteins

The immunomodulatory activities of twenty-two edible mushroom proteins were compared using the homogenized computational modal length method. Three vectors Xi (i=1,2,3; Xi represents the ratio of the three original indexes of phagocytosis, NO, and ROS to the corresponding control group, respectively) were set, and the top six most immunoreactive proteins from the precious edible mushroom and common edible mushroom proteins were selected by calculating ||X||^2^ according to the mode length formula as follows.


$$||X|{|}^{2}={\left[X_{1}^{2}/(X_{1}+X_{2}+X_{3})\right]}^{2}+{\left[X_{2}^{2}/(X_{1}+X_{2}+X_{3})\right]}^{2}+{\left[X_{3}^{2}/(X_{1}+X_{2}+X_{3})\right]}^{2}$$

### Effects of the release of the active protein RAW 264.7 immune factors IL-1β, IL-6, IFN-γ, and TNF-α from edible bacteria

Macrophages in the logarithmic growth phase were inoculated in 6-well plates, and 2 mL of cell culture medium was added to each well; samples of different concentrations were added and incubated in a cell culture incubator containing 5% CO2 for 24 h. The supernatant was extracted (40, 41). The supernatant was collected. The content of IL-1β, IL-6, TNF-α, and IFN-γ in the supernatant was determined by referring to the ELISA kit instructions. Among them, the group with 1 μg/mL lipopolysaccharide addition was used as a positive control.

### Comparison of the immune activity of proteins of precious edible mushrooms and common edible mushrooms

The immunomodulatory activities of the screened proteins of precious edible mushrooms and common edible mushrooms were compared using the homogenized computational modal length method. Four vectors Xi (i=1,2,3,4; Xi represents the ratio of the original indexes of IL-1β, IL-6, IFN-γ, and TNF-α to the corresponding control group, respectively) were set, and ||X||2 was calculated according to the mode length formula as follows.


$$\begin{array}{c} ||X|{|}^{2}={\left[X_{1}^{2}/(X_{1}+X_{2}+X_{3}+X_{4})\right]}^{2}+{\left[X_{2}^{2}/(X_{1}+X_{2}+X_{3}+X_{4})\right]}^{2}+{\left[X_{3}^{2}/(X_{1}+X_{2}+X_{3}+X_{4})\right]}^{2}\\ +{\left[X_{4}^{2}/(X_{1}+X_{2}+X_{3}+X_{4})\right]}^{2} \end{array}$$

## Results

### Composition analysis of edible mushrooms

The protein content of 22 edible fungi was analyzed by the method of Casteldahl. The relative dry weight content ranged from 17.42% to 41.21%, and the protein content of *O. sinensis*, *C. militaris*, and *A. aegerita* were relatively high. More than 35%; The protein content of *G. frondosa*, T. Matsutake Singer, and *C. versicolor* were all lower than 20%, which may be due to the high content of polysaccharide, lignin, and uronic acid (**Table 1**).

**Table 1:** Protein content of different edible mushrooms (n=6).

Edible mushrooms	Protein(%)
***Russula alutacea***	41.21±0.56
***Volvariella volvacea***	38.65±1.14
***Agrocybe aegerita***	38.65±0.13
***Morchella esculenta***	36.79±0.42
***Boletus***	35.52±0.25
***Tuber melanosporum***	29.60±0.08
***Agaricus bisporus***	29.23±0.21
***Pleurotus eryngii***	28.48±0.47
***Ganoderma lucidum***	27.48±0.03
***Ophiocordyceps sinensis***	26.31±0.05
***Cordyceps militaris***	25.65±0.49
***Lentinula edodes***	25.48±0.31
***Hypsizygus marmoreus***	24.67±0.21
***Flammulina velutipes***	24.40±0.31
***Phellinus igniarius***	24.02±0.16
***Pleurotus ostreatus***	22.67±0.28
***Termitornyces albuminosus***	21.52±0.16
***Inonotus obliquus***	20.44±0.18
***Hericium erinaceus***	20.23±0.06
***Grifola frondosa***	18.44±0.18
***Tricholoma matsutake Singer***	18.40±0.26
***Coriolus versicolor***	17.42±0.11

The present data were expressed the mean ± SD.

Twenty-two edible fungal proteins were extracted by alkali solubilization and acid precipitation method, and ammonium sulfate precipitation method with relative dry weight yields of 1.1%-3.4%. The protein content of the method was 60.06%-75.82%, all above 60.00%, indicating that the main components of the samples were proteins, with CMP proteins reaching 75.82%, and as polysaccharides in fungi are the main interfering substances in protein samples, the polysaccharide content was 4.42%-9.69%, with the highest VVP polysaccharide content of 9.69%, and the other components were cellulose, ash, water, glyoxylate, lignin, and unknown fractions, which contained extremely low concentrations of polysaccharide contamination due to the sample concentration of 25-200 μg/mL in the activity experiments, thus excluding their interference with protein activity. The glyoxylate and lignin contents of the twenty-two polysaccharides were low, with the lowest RAP glyoxylate content of 0.79% and the highest ABP glyoxylate content of 1.62%; the lowest VVP lignin content of 0.41% and the highest GFP plasmalogen content of 2.14%, where the cellulose content was 5.79%-11.04%, the ash content was 2.69%-7.69%, and the moisture content was 1.15%-3.65%. At the same time, the samples also contained some unknown components, which may be undetectable organic compounds such as pigments, with the highest unknown component of 11.45% for AAP and the lowest unknown component of 2.24% for PEP, indicating a relatively complex composition (**Table 2**).

**Table 2:** Composition of proteins (n=6).

	Protein	Polysaccharide	Cellulose	Mositure	Ash	uronic acid	Lignin	Unknown
**CMP**	75.82±0.10	8.61±0.06	6.47±0.1	1.91±0.11	2.69±0.19	1.13±0.11	1.81±0.01	4.13±0.35
**PEP**	73.35±0.14	5.72±0.35	8.87±0.11	2.28±0.06	5.71±0.18	1.00±0.15	0.83±0.06	2.24±0.39
**POP**	73.05±0.10	5.75±0.18	9.55±0.33	2.34±0.06	3.35±0.10	1.04±0.03	0.51±0.04	4.41±0.26
**TAP**	69.97±1.26	8.74±0.20	6.45±0.28	1.56±0.02	5.79±0.24	1.23±0.16	1.16±0.07	5.11±0.99
**LEP**	69.39±0.30	5.86±0.22	10.13±0.39	2.09±0.08	3.64±0.33	1.42±0.04	1.91±0.05	5.56±0.89
**ABP**	69.23±3.01	4.42±0.16	6.01±0.20	1.75±0.05	6.69±0.19	1.62±0.07	1.74±0.09	8.53±3.22
**VVP**	69.22±0.23	9.69±0.24	7.89±0.08	1.61±0.03	5.45±0.30	0.86±0.04	0.41±0.03	4.86±0.52
**HEP**	68.78±0.12	7.38±0.16	7.92±0.35	1.54±0.12	5.39±0.20	0.93±0.07	0.46±0.05	7.60±0.81
**HMP**	66.79±0.10	5.14±1.00	5.79±0.19	2.34±0.23	6.82±0.13	1.31±0.30	1.96±0.02	9.85±1.51
**CVP**	64.30±0.37	8.82±0.17	9.50±0.31	1.19±0.03	3.34±0.16	1.07±0.16	1.95±0.04	9.82±0.58
**OSP**	63.82±0.71	6.78±0.13	9.73±0.36	2.16±0.04	5.37±0.19	1.27±0.06	0.48±0.03	10.39±0.73
**PIP**	62.37±0.14	9.67±0.31	8.82±0.31	2.37±0.08	4.62±0.31	1.09±0.06	1.04±0.05	10.01±0.60
**GLP**	62.35±0.88	9.08±0.42	9.19±0.34	1.37±0.06	6.52±0.20	0.81±0.06	1.87±0.03	8.80±0.55
**MEP**	62.21±0.48	9.50±0.35	10.04±0.49	1.16±0.02	5.72±0.14	0.82±0.03	0.76±0.06	10.04±1.07
**RAP**	62.02±0.37	8.74±0.31	10.69±0.17	1.15±0.01	5.56±0.22	0.79±0.02	0.48±0.08	10.56±0.44
**IOP**	61.80±0.31	8.94±0.51	9.26±0.36	1.53±0.02	6.62±0.17	1.05±0.04	1.01±0.84	9.78±0.92
**BP**	61.16±0.32	9.22±0.18	9.44±0.61	3.01±0.14	5.76±0.38	0.88±0.02	1.21±0.04	9.31±0.88
**AAP**	60.92±0.47	8.74±0.18	10.16±0.39	2.16±0.22	3.59±0.15	1.25±0.07	1.72±0.01	11.45±0.83
**TMP**	60.53±0.84	6.82±0.36	9.97±0.26	2.46±0.07	7.57±0.21	1.14±0.03	0.50±0.07	11.00±0.79
**FVP**	60.40±0.86	8.10±0.13	11.04±0.43	3.40±0.44	6.40±0.27	1.01±0.14	1.44±0.03	8.20±1.27
**TMSP**	60.30±0.84	8.76±0.24	10.68±0.28	3.65±0.45	7.53±0.39	0.87±0.03	1.26±0.03	6.94±0.16
**GFP**	60.06±2.16	8.86±0.44	7.66±0.29	3.07±0.21	7.69±0.09	1.03±0.09	2.14±0.03	9.49±1.66

The present data were expressed the mean ± SD.

### Edible mushroom proteins enhance RAW 264.7 proliferation

**Figure 1:**
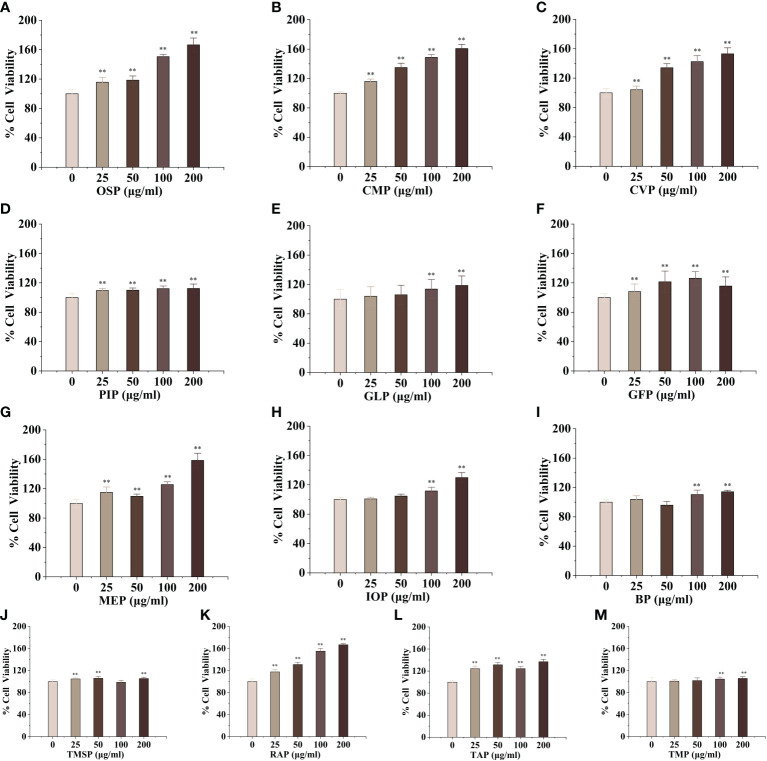
Proliferative effects of thirteen rare edible mushroom proteins on RAW 264.7 cells. OSP **(A)**, CMP **(B)**, CVP **(C)**, PIP **(D)**, GLP **(F)**, MEP **(G)**, IOP **(H)**, BP **(I)**, TMSP **(J)**, RAP **(K)**, TAP **(L)**, and TMP **(M)** on the proliferation of RAW 264.7 macrophages. Data are expressed as mean ± SD (n=5). **P < 0.01, compared with the control group.

**Figure 2:**
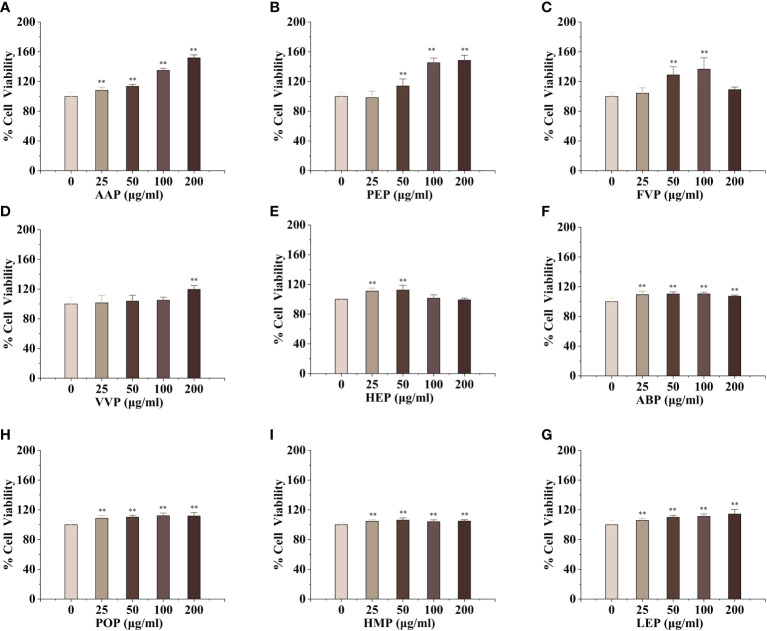
Proliferative effects of nine common edible mushroom proteins on RAW 264.7 cells. AAP **(A)**, PEP **(B)**, FVP **(C)**, VVP **(D)**, HEP **(E)**, ABP **(F)**, LEP **(G)**, POP **(H)** and HMP **(I)** on the proliferation of RAW 264.7 macrophages. Data are expressed as mean ± SD (n = 5). **P < 0.01; compared with the control group.

Macrophages are the main component of the monocyte-macrophage system, which not only initiate the innate immune (non-specific immune) response but also participate in the cellular immune (specific immune) response. Activated macrophages can engulf a variety of pathogenic microorganisms that invade the body, process, and present antigens, as well as synthesize and secrete a variety of chemokines and cytokines to enhance the body’s resistance to infection and immune defense. To evaluate the activation effect of different concentrations of edible mushroom proteins on mouse macrophages RAW264.7, this experiment used the cck-8 method to detect the survival rate of mouse macrophages co-cultured with certain concentrations of proteins The experimental results showed that, compared to the blank control, CMP, AAP, CVP, OSP, PIP, RAP, TAP, TMP, VVP, MEP, BP, FVP, PEP, TMSP, IOP, GFP, LEP, POP, HMP, ABP, VVP, and HEP proteins were found to be less toxic at the four measured All twenty-two edible mushroom proteins showed significant dose effects, and the value-added rate of macrophages changed with the change of concentration. MEP, RAP, TAP, PEP, POP, and LEP increased with increasing concentrations, and the value-added rate of macrophages reached the highest at the concentration of 200 μg/mL, which was highly significantly different from that of the blank control group, and the proliferation of macrophages by OSP, CMP, CVP, AAP, MEP, RAP, TAP, PEP, POP, LEP, and HMP reached 166.57 ± 9.29%, 160.75 ± 5.67%, 151.71 ± 4.05%, 153.11 ± 8.24%, 158.51 ± 9.62%, 166.72 ± 1.99%, 137.01 ± 4.26%, 148.42 ± 6.93%, 111.67 ± 4.54%, 114.30 ± 6.30%, and 148.42 ± 6.93%; PIP proliferated 112.29 ± 6.05% of macrophages at 200 μg/mL; TMSP proliferated 105.89 ± 3.49% of macrophages at 50 μg/mL; HEP proliferated 105.89 ± 3.49% of macrophages at low concentrations (25 and 50 μg/mL), with a highly significant difference in the proliferation rate of macrophages at 50 μg/mL. There were no significant differences at high concentrations (100 and 200 μg/mL); GLP, IOP, BP, TMP, and VVP were not significantly different from the blank control at low concentrations (25 and 50 μg/mL) and significantly increased the macrophage proliferation rate at 200 μg/mL, reaching 118.64 ± 4.75%, 129.88 ± 3.49%, respectively. The proliferation of GFP, FVP, ABP, and HMP on macrophages showed a bell-shaped effect on the concentration and the maximum value-added rates of GFP, FVP, and ABP at 100 μg/mL 115.60 ± 12.46%, 136.56%, 136.56%, and 136.56%, respectively. 12.46%, 136.56 ± 15.31%, 110.22 ± 2.40%, and HMP at 50 μg/mL with a maximum value-added rate of 106.13 ± 3.11%. The survival rate of macrophages was significantly higher, indicating that the concentration is within the safe dose range selected, and the concentration range can be used for subsequent experiments. (**Figures 1**, **2**)

### Mushroom protein consumption enhances RAW 264.7 phagocytosis

**Figure 3:**
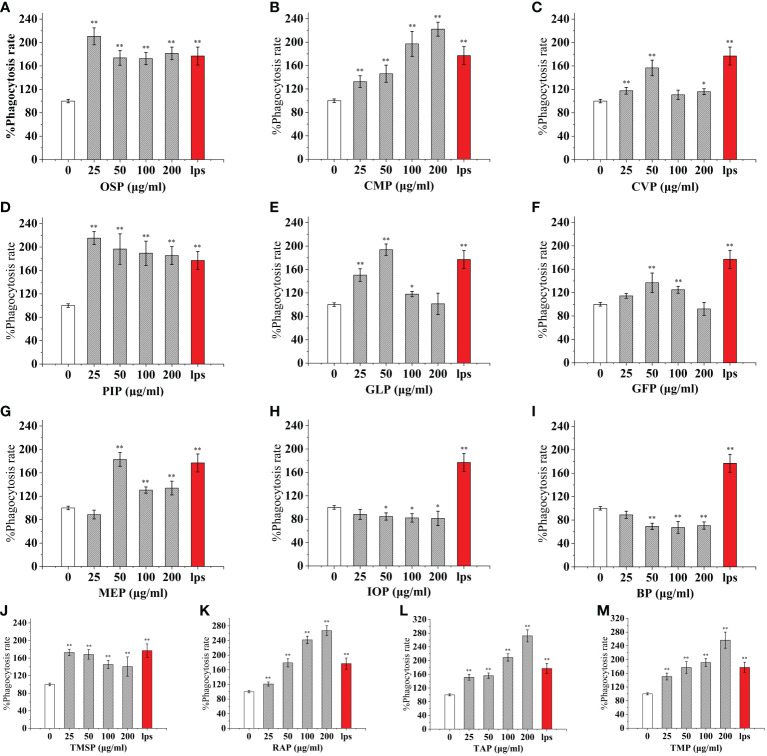
Phagocytosis of thirteen rare edible mushroom proteins on RAW 264.7 cells. OSP **(A)**, CMP **(B)**, CVP **(C)**, PIP **(D)**, GLP **(E)**, GFP **(F)**, MEP **(G)**, IOP **(H)**, BP **(I)**, TMSP **(J)**, RAP **(K)**, TAP **(L)**, and TMP **(M)** on the phagocytosis of RAW 264.7 cells. Data are expressed as mean ± SD (n = 5). *P < 0.05, **P < 0.01, compared with the control group.

**Figure 4:**
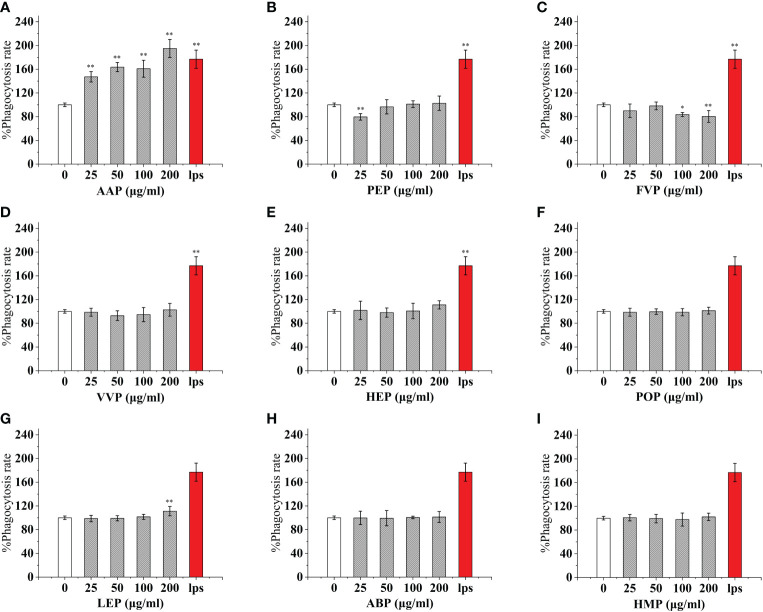
Phagocytosis of nine common edible mushroom proteins on RAW 264.7 cells. AAP **(A)**, PEP **(B)**, FVP **(C)**, VVP **(D)**, HEP **(E)**, POP **(F)**, LEP **(G)**, ABP **(H)** and HMP **(I)**) on the phagocytosis of RAW 264.7 macrophages. Data are expressed as mean ± SD (n = 5). *P <0.05, **P <0.01, compared with the control group.

As an important component of the body’s natural immune defense, macrophages can engulf pathogens and senescent cells in the body. When macrophages are activated, their cell volume increases, their acid hydrolase activity increases, and their ability to kill bacteria and digest and swallow foreign bodies are enhanced. At the same time, activated macrophages can also promote neutrophils and lymphocytes to tend to the wound and participate in the inflammatory response synergistically to promote wound healing. Compared with the control, The effect of CMP, AAP, IOP, RAP, TAP, TMP, LEP increased with increasing concentration and the most phagocytic capacity at 200 μg/mL was 125.57%, 110.38%, 81.25%, 150.86%, 125.57%, 144.94%, 111.20% of the positive control, respectively.OSP, PIP, TMSP were the most phagocytosed at 25 μg/mL being 119.16%, 121.60%, 98.11% of the positive control.CVP and BP at 100 μg/mL were not significantly different from the control. The effect showed a significant “ bell-type” dose dependence, with the strongest phagocytosis at 50 μg/mL being 104.78% and 67.18% of the positive control. The effects of GLP, GFP, MEP, CVP, BP, and GLP increased with increasing concentrations in the range of 0-50 μg/mL, and inhibited cell phagocytosis due to doses at concentrations of 100 and 200 μg/mL, with the strongest phagocytosis at 50 μg/mL being 109.55%, 77.27%, 136.45%, 104.78%, and 67.18%.PEP, VVP, HEP, ABP, POP, HMP, and FVP showed no significant difference compared to the control at 25-200 μg/mL. (**Figures 3**, **4**)

### Mushroom protein consumption enhances the secretion of reactive oxygen species by RAW 264.7

Reactive oxidative species (ROS) are oxygen radicals and their derivatives that are directly or indirectly transformed by oxygen, which is generated mainly through the cell itself, exogenous environment, or as by-products of other biological reactions (42). When the body is in acute or chronic inflammation, phagocytes, such as macrophages and neutrophils, are activated, leading to enhanced respiration, increased oxygen consumption, and the production of ROS, which can be involved in the synthesis of a series of inflammatory factors or increase the phagocytic capacity of cells to kill bacteria and other exogenous substances (43).To determine the effect of different concentrations of edible proteins (0-200 μg/mL) on ROS secretion in macrophages, a fluorescent probe DCFH-DA was used in this study to determine the changes in ROS secretion in cells after 24 h of edible protein treatment. When DCFH-DA diffuses into the cell, it can be hydrolyzed and deacetylated by esterase into non-fluorescent 2’,7’-dichlorobis(hydrogen fluorescein) (DCFH), which can be oxidized by ROS into high-intensity, green fluorescent 2’,7’-dichlorobis(hydrogen fluorescein) (DCFH). DCFH can be oxidized by ROS to 2’,7’-dichlorofluorescein (DCF) with high intensity and green fluorescence, and the fluorescence intensity was positively correlated with the intracellular ROS level (44).

**Figure 5:**
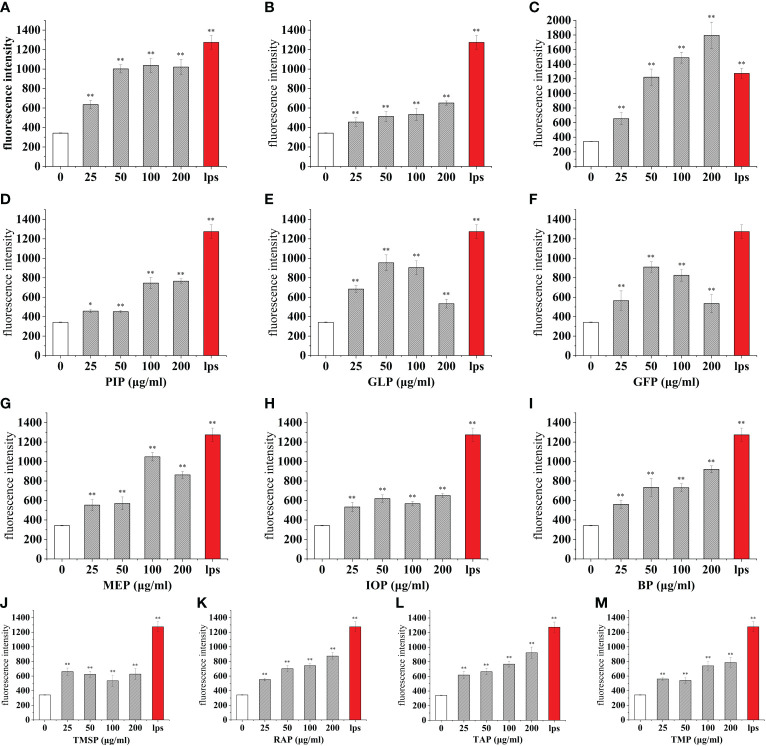
Effect of different edible mushroom protein treatments on ROS production in RAW 264.7 cells. OSP **(A)**, CMP **(B)**, CVP **(C)**, PIP **(D)**, GLP **(E)**, GFP **(F)**, MEP **(G)**, IOP **(H)**, BP **(I)**, TMSP **(J)**, RAP **(K)**, TAP **(L)** and TMP **(M)** on RAW 264.7 cells for ROS production. Data are expressed as mean ± SD (n = 5). *P < 0.05 and **P < 0.01 compared to the control group.

**Figure 6:**
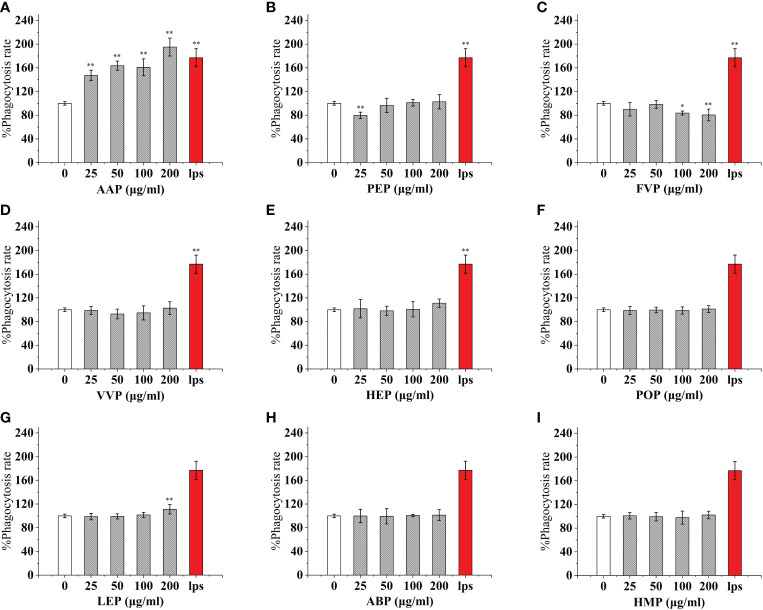
Effect of different edible mushroom protein treatments on ROS production in RAW 264.7 cells AAP **(A)**, PEP **(B)**, FVP **(C)**, VVP **(D)**, HEP **(E)**, POP **(F)**, LEP **(G)**, ABP **(H)** and HMP **(I)** on RAW 264.7 cells for ROS production. Data are expressed as mean ± SD (n=5). *P < 0.05 and **P < 0.01 compared to the control group.

The mean cellular fluorescence intensity (ROS production) was 342.09 ± 3.62 for the blank control and 1274.31 ± 69.53 for the positive control LPS, while the mean cellular fluorescence intensity of LPS (100 ng/mL) and a certain concentration of edible mushroom protein was increased compared to the blank control. Among them, the cellular ROS secretion levels reached 51.85%, 54.89%, and 58.61% of the positive control LPS after the addition of 25 μg/mL of TMSP, FVP, and VVP treatments, respectively. After the addition of 50 μg/mL of AAP, GLP, and GFP, the cellular ROS secretion levels reached 47.85%, 74.86%, and 71.42% of the positive control LPS, respectively. After the addition of 100 μg/mL of OSP, MEP, PEP, and ABP, the cellular ROS secretion levels reached 81.48%, 82.33%, 59.59%, and 58.74% of the positive control LPS, respectively.After treatment with 200 μg/mL of CMP, CVP, PIP, IOP, BP, RAP, TAP, TMP, HEP, POP, LEP, HMP, respectively, the cellular ROS secretion levels reached 51.09%, 140.93%, 60.05%, 51.11%, 72.20%, 68.58%, 72.48%, 61.65%, 51.35%, 60.14%, 72.21%, 69.11% of the positive control LPS, respectively.Among them, OSP, MEP, and CVP promoted the higher cellular secretion of ROS secretion, all reaching 80%, and AAP promoted the lower cellular secretion of ROS secretion, below 50%. the highest ROS fractions all reached highly significant levels (p < 0.01). (**Figures 5**, **6**)

### Mushroom protein consumption enhances nitric oxide secretion by RAW 264.7

**Figure 7:**
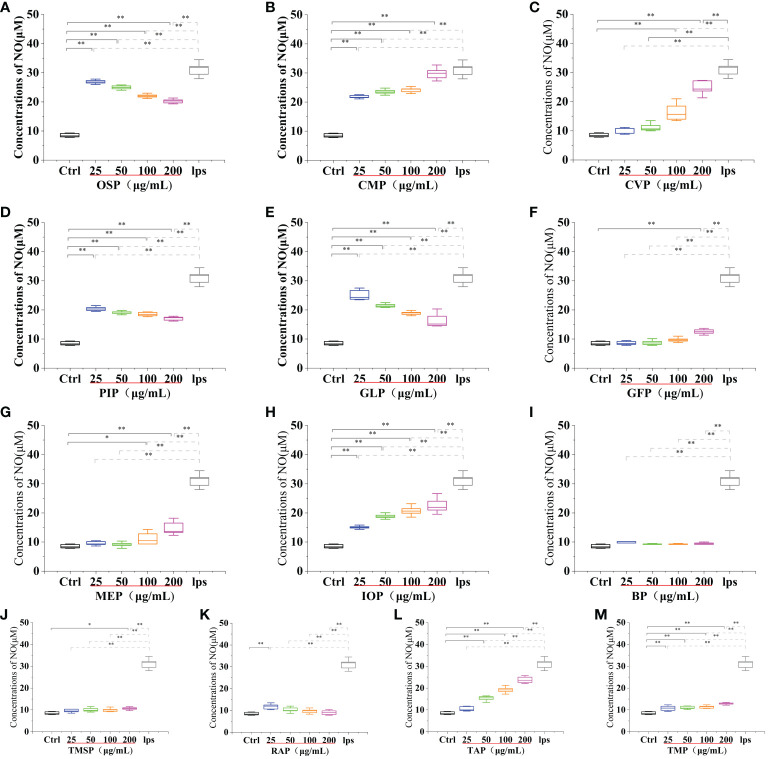
Effect of different edible mushroom protein treatments on NO production in RAW 264.7 cells. OSP **(A)**, CMP **(B)**, CVP **(C)**, PIP **(D)**. GLP **(E)**, GFP **(F)**. MEP **(G)**, IOP **(H)**, BP **(I)**, TMSP **(J)**, RAP **(K)**, TAP **(L)** and TMP **(M)** on RAW 264.7 cells for NO production. Data are expressed as mean ± SD (n = 5). *P < 0.05 and **P < 0.01 compared to the control group.

**Figure 8:**
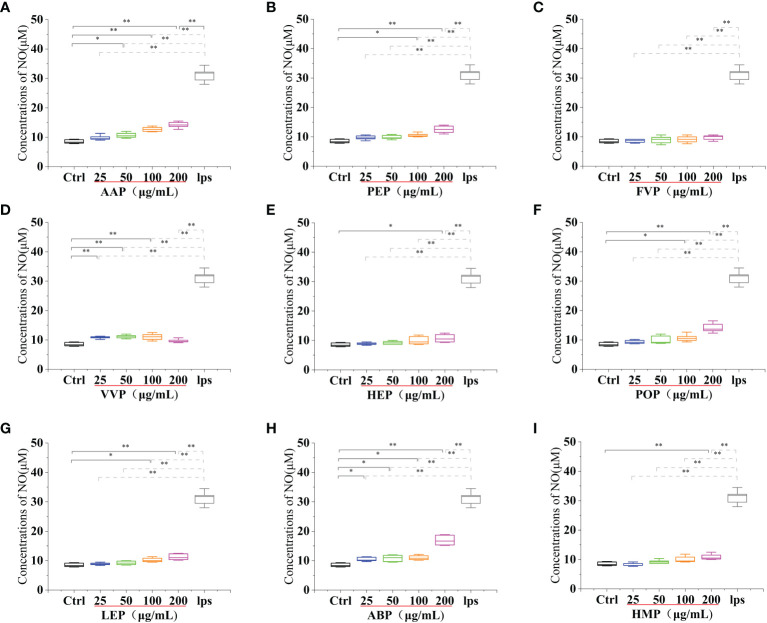
Effect of different edible mushroom protein treatments on NO production in RAW 264.7 cells AAP **(A)**, PEP **(B)**, FVP **(C)**, VVP **(D)**, HEP **(E)**, POP **(F)**, LEP **(G)**, ABP **(H)** and HMP **(I)** on RAW 264.7 cells for NO production. Data are expressed as mean ± SD (n = 5). *P <0.05 and **P < 0.01 compared to the control group.

Activated macrophages can secrete a series of chemokines and cytokines, which play a crucial role in inactivating the adaptive immune response of the body and regulating various immune responses. Among them, nitric oxide (NO) is a reactive nitrogen intermediate that belongs to free radicals with oxidative properties. NO is usually considered to have a strong killing ability, can lead to apoptosis of tumor cells, and can act as an intracellular messenger molecule to mediate various biological responses (45). CMP, AAP, CVP, GFP, MEP, IOP, TMSP, TAP, TMP, PEP, HEP, POP, LEP, ABP, and HMP, at concentrations of 25-200 μg/mL significantly stimulated NO secretion from RAW 264.7 cells, and showed a significant dose-effect relationship, with increasing protein concentration, NO secretion gradually The amount of NO secretion increased with the increase of protein concentration. After stimulating the cells with a 200 μg/mL protein sample for 24 h, the NO secretion level reached a significant difference (p < 0.01) compared with the blank control group, and also reached 95.57%, 45.51%, 79.39%, 40.16%, 47.43%, 72.5%, 34.07% of the positive control group 31.20 ± 2.25 μM compared with the LPS positive treatment group. OSP, GLP, RAP, and PIP were able to significantly stimulate NO secretion from RAW 264.7 cells at concentrations of 25-200 μg/mL and showed a significant dose-effect relationship. The NO secretion levels reached 26.90 ± 0.64 μM, 25.13 ± 1.64 μM, 11.80 ± 1.17 μM and 20.40 ± 0.71 μM after 24 h of stimulation with 25 μg/mL protein samples, and 11.16 ± 0.57 μM after 24 h of stimulation with 50 μg/mL VVP samples, respectively. The levels of NO secretion reached 11.16 ± 0.57 μM after 24 h of stimulation with 50 μg/mL of VVP samples, which were significantly different from the blank control group (p < 0.01) and 86.21%, 80.54%, 37.82%, 65.38%, and 35.76% of the positive control group, respectively. The significant increase in NO secretion indicated that the protein may have strong immunostimulatory and tumor cell growth inhibitory activities. No significant increase in NO secretion indicated that BP and FVP did not have a strong immunostimulatory effect and tumor cell growth inhibitory activity. The highest NO secretion fractions reached highly significant levels (p < 0.01). (**Figures 7**, **8**)

### Comparison of the immune activity of twenty-two edible mushroom proteins

A comprehensive analysis of the effects of the above twenty-two edible mushroom proteins on NO secretion, phagocytosis, and ROS secretion by mouse macrophages revealed that the proteins of different edible mushrooms showed different degrees of promoting immune activity of macrophages. The immunomodulatory activities of twenty-two edible mushroom proteins were compared using a mathematical method of homogenization to calculate the modal lengths to select the best edible mushroom protein with the best immune activity. The paper firstly standardized the three original indices of NO secretion, phagocytosis, and ROS secretion, and then homogenized the formula of mode length to calculate ||X||^2^, and analyzed the variance of ||X||^2^ to reveal the differences of immune activity of twenty-two edible bacteria proteins.

The immunoreactivity of the twenty-two edible mushroom proteins was compared as shown in the table (**Table 3**), and the ||X||^2^ of the twenty-two proteins in the concentration range of 25-200 μg/mL was the largest at 200 μg/mL for CMP, which was 9.98 ± 1.31. The ||X||^2^ of the twenty-two proteins at each concentration was subjected to ANOVA with them, and the results showed that, except for CVP at 200 μg/mL || X||^2^ of 9.74 ± 1.16 were not significantly different, all of them were highly significantly different (**P<0.01). In summary, among the twenty-two edible mushroom proteins, the immunomodulatory activities were CMP, CVP, IOP, TAP, MEP, OSP, BP, RAP, GLP, GFP, TMP, PIP, VVP, PEP, POP, ABP, FVP, TMSP, HEP, AAP, LEP, HMP in descending order, and from the table, it can be seen that the immunomodulatory activities of cherished edible mushrooms The immunomodulatory activities of cherished edible mushrooms were significantly higher than those of common edible mushrooms. The top six species with the best immune effect were selected for further experiments for the rare edible mushrooms CMP, CVP, IOP, TAP, MEP, OSP, and the common edible mushrooms VVP, PEP, POP, ABP, FVP, HEP, respectively.

**Table 3:** Comparison of the immune activity of twenty-two edible mushroom proteins (n=5).

Groups	Concentration(μg/mL)	X_1_	X_2_	X_3_	||X||^2^
**OSP**	25	2.11±0.14	3.17±0.26	1.86±0.11	2.66±0.45**
	50	1.74±0.13	2.94±0.19	2.93±0.12	2.74±0.29**
	100	1.73±0.10	2.59±0.16	3.03±0.21	2.60±0.31**
	200	1.81±0.11	2.39±0.18	2.98±0.20	2.42±0.30**
**CMP**	25	1.33±0.10	3.97±0.06	1.33±0.12	5.82±0.28**
	50	1.46±0.15	4.29±0.13	1.50±0.14	6.67±0.74**
	100	1.97±0.21	4.40±0.07	1.56±0.18	6.33±0.44**
	200	2.22±0.12	5.43±0.28	1.90±0.05	**9.98±1.31**
**AAP**	25	1.47±0.09	1.13±0.09	1.26±0.04	0.61±0.04**
	50	1.64±0.08	1.23±0.11	1.78±0.21	0.94±0.16**
	100	1.61±0.14	1.46±0.10	1.17±0.28	0.79±0.08**
	200	1.95±0.15	1.63±0.17	1.47±0.04	1.05±0.14**
**CVP**	25	1.18±0.06	1.13±0.12	1.91±0.23	1.00±0.29**
	50	1.57±0.13	1.29±0.14	3.45±0.13	3.82±0.38**
	100	1.11±0.08	1.90±0.32	4.35±0.21	7.08±1.39**
	200	1.16±0.05	2.83±0.15	5.25±0.22	9.74±1.16
**PIP**	25	2.15±0.11	2.34±0.12	1.34±0.05	1.62±0.19**
	50	1.96±0.26	2.18±0.12	1.32±0.03	1.41±0.13**
	100	1.89±0.21	2.13±0.14	2.18±0.17	1.50±0.15**
	200	1.85±0.15	1.96±0.11	2.24±0.08	1.43±0.09**
**GLP**	25	1.50±0.11	2.34±0.12	2.00±0.11	2.21±0.08**
	50	1.94±0.10	2.18±0.12	2.79±0.26	2.20±0.28**
	100	1.18±0.04	2.13±0.14	2.65±0.23	2.06±0.35**
	200	1.01±0.18	1.96±0.11	1.56±0.13	1.06±0.37**
**GFP**	25	1.14±0.04	0.99±0.08	1.65±0.28	0.77±0.28**
	50	1.37±0.17	1.01±0.09	2.66±0.13	2.18±0.32**
	100	1.25±0.06	1.12±0.08	2.41±0.17	1.68±0.27**
	200	0.92±0.11	1.44±0.09	1.56±0.27	0.77±0.21**
**MEP**	25	0.89±0.07	1.10±0.10	1.86±0.11	0.96±0.12**
	50	1.83±0.12	1.04±0.09	2.93±0.12	2.57±0.31**
	100	1.31±0.05	1.29±0.23	3.03±0.21	2.89±0.46**
	200	1.34±0.12	1.69±0.17	2.98±0.20	2.54±0.38**
**IOP**	25	0.88±0.09	2.81±0.10	1.56±0.14	2.52±0.21**
	50	0.85±0.06	3.52±0.13	1.81±0.11	4.32±0.39**
	100	0.82±0.07	3.86±0.12	1.66±0.08	5.76±0.52**
	200	0.81±0.12	4.21±0.33	1.90±0.07	6.94±1.45**
**BP**	25	0.89±0.06	1.13±0.03	1.64±0.14	0.72±0.13**
	50	0.69±0.05	1.07±0.04	2.15±0.27	1.54±0.49**
	100	0.67±0.10	1.06±0.04	2.14±0.11	1.51±0.25**
	200	0.71±0.06	1.09±0.05	2.69±0.12	2.70±0.30**
**TMSP**	25	1.73±0.07	1.10±0.13	1.93±0.13	1.10±0.13**
	50	1.68±0.11	1.16±0.06	1.82±0.11	0.98±0.07**
	100	1.46±0.09	1.14±0.06	1.57±0.23	0.74±0.16**
	200	1.41±0.22	1.22±0.08	1.83±0.21	0.93±0.19**
**RAP**	25	1.21±0.06	1.35±0.14	1.62±0.06	0.72±0.05**
	50	1.79±0.11	1.19±0.15	2.05±0.10	1.21±0.17**
	100	2.42±0.10	1.11±0.12	2.17±0.11	1.80±0.14**
	200	2.67±0.13	1.04±0.14	2.55±0.12	2.43±0.28**
**TAP**	25	1.51±0.08	1.94±0.18	1.81±0.15	1.12±0.15**
	50	1.56±0.08	2.83±0.16	1.94±0.12	2.12±0.23**
	100	2.09±0.11	3.56±0.26	2.24±0.11	3.34±0.52**
	200	2.72±0.18	4.4±0.16	2.70±0.22	5.04±0.35**
**TMP**	25	1.51±0.1	1.24±0.13	1.64±0.06	0.77±0.07**
	50	1.77±0.17	1.27±0.12	1.58±0.12	0.89±0.13**
	100	1.91±0.11	1.31±0.05	2.16±0.19	1.35±0.19**
	200	2.56±0.24	1.47±0.04	2.29±0.17	1.92±0.36**
**PEP**	25	0.80±0.05	1.12±0.08	1.69±0.19	0.81±0.23**
	50	0.97±0.12	1.13±0.03	1.98±0.18	1.11±0.28**
	100 200	1.01±0.06	1.21±0.06	2.22±0.11	1.40±0.17**
		1.03±0.12	1.44±0.16	1.54±0.18	0.71±0.15**
**FVP**	25	0.90±0.11	0.99±0.08	2.05±0.12	1.25±0.20**
	50	0.98±0.07	1.03±0.14	1.41±0.17	0.53±0.13**
	100	0.84±0.03	1.05±0.14	1.40±0.13	0.54±0.08**
	200	0.80±0.10	1.12±0.11	1.38±0.18	0.55±0.12**
**VVP**	25	0.99±0.07	2±0.1	1.47±0.15	1.10±0.11**
	50	0.93±0.08	2.06±0.14	2.18±0.34	1.65±0.31**
	100	0.95±0.12	2.04±0.17	1.86±0.15	1.32±0.23**
	200	1.03±0.11	1.79±0.08	1.96±0.10	1.16±0.06**
**HEP**	25	1.02±0.15	1.02±0.05	1.21±0.03	0.42±0.02**
	50	0.98±0.08	1.06±0.08	1.29±0.09	0.45±0.05**
	100	1.01±0.13	1.15±0.15	1.63±0.06	0.71±0.05**
	200	1.11±0.07	1.24±0.16	1.91±0.08	0.97±0.08**
**POP**	25	0.99±0.07	1.07±0.09	1.63±0.05	0.69±0.04**
	50	1.00±0.05	1.15±0.16	1.69±0.04	0.76±0.04**
	100	0.99±0.06	1.23±0.13	1.87±0.04	0.93±0.05**
	200	1.01±0.06	1.63±0.17	2.01±0.05	1.15±0.04**
**LEP**	25	0.99±0.05	1.02±0.06	1.21±0.03	0.41±0.02**
	50	0.99±0.04	1.06±0.09	1.29±0.09	0.45±0.05**
	100	1.02±0.04	1.18±0.13	1.28±0.08	0.48±0.07**
	200	1.11±0.08	1.3±0.15	1.51±0.06	0.64±0.06**
**ABP**	25	1.00±0.11	1.2±0.09	1.70±0.16	0.79±0.18**
	50	0.99±0.13	1.24±0.14	1.98±0.15	1.10±0.22**
	100	1.01±0.02	1.26±0.09	2.19±0.05	1.34±0.05**
	200	1.01±0.09	1.94±0.21	1.62±0.11	1.09±0.25**
**HMP**	25	1.01±0.05	0.97±0.09	1.21±0.03	0.40±0.03**
	50	0.99±0.07	1.06±0.1	1.29±0.09	0.46±0.05**
	100	0.98±0.11	1.16±0.11	1.28±0.08	0.47±0.06**
	200	1.02±0.06	1.25±0.11	1.39±0.08	0.55±0.06**

The present data were expressed the mean ± SD.

^**^means significant difference between 200 μg/mL of CMP group, p < 0.01.

### Mushroom protein consumption enhances the secretion of cytokines IL-1β, IL-6, TNF-α, and IFN-γ in RAW 264.7

**Figure 9:**
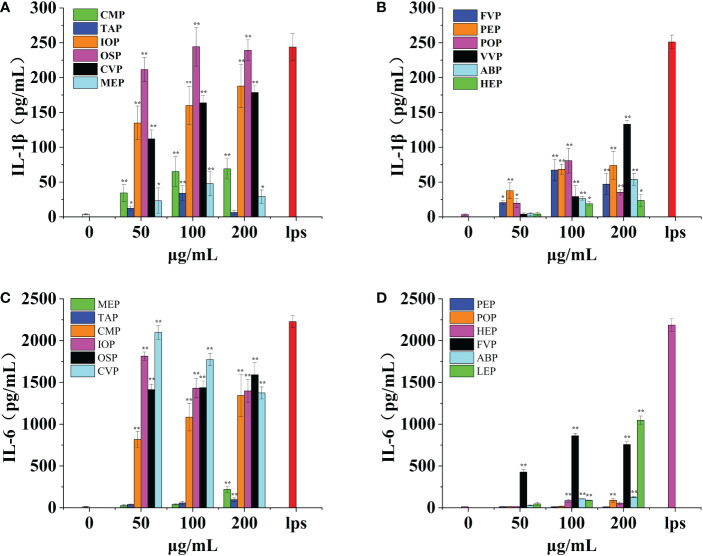
Effect of six rare edible mushrooms on IL-1ß production in RAW 264.7 macrophages **(A)**. Effect of six common edible bacteria on IL- 1ß production in RAW 264.7 macrophages **(B)**, Effect of six rare edible bacteria on IL-6 production in RAW 264.7 macrophages **(C)**, Effect of six common edible bacteria on IL-6 production in RAW 264.7 macrophages **(D)**. *p < 0.05, **p < 0.01 indicates statistically significant difference compared to positive control group.

Macrophages have a variety of receptors distributed on their surface that can bind different proteins and produce various effector molecules when activated. IL-1β is one of the effector molecules produced by macrophages and can be involved in fever by inducing an acute phase protein response and also induces tumor cell death *via* NK cells, acting in conjunction with IL-12 and IFN-γ. IL-1β acts as a key pro-inflammatory cytokine. CMP, CVP, IOP, TAP, MEP, OSP, FVP, PEP, POP, VVP, ABP, and HEP, all promoted IL-1β production by macrophages in a significant dose-dependent manner, reaching a minimum of 16.45% of positive controls and a maximum of 99.70% of positive controls.CMP, PEP, VVP, ABP, HEP, IOP, and CVP had the strongest effect at a concentration of 200 μg/mL, when the production of IL-1β was 30.62%, 29.40%, 52.96%, 21.44%, 9.37%, 70.22% and 70% of the positive control, respectively. TAP, FVP, POP, OSP, and MEP had the strongest effect at a concentration of 100 μg/mL, showing a clear bell shape, when IL-1β production was 13.39%, 26.75%, 32.10%, 99.70%, and 16.45% of the positive control group, respectively; among them, OSP, IOP, and CVP promoted IL-1β production by macrophages with the best effect all reaching 70%, and HEP, TAP, and MEP promoted IL-1β production by macrophages with poorer effect below 20%. (**Figures 9A, B**)

IL-6 is a pluripotent cytokine that not only affects the immune system but also plays a role in other biological systems and many physiological events, such as the regulation of cell growth and gene activation. OSP, CMP, CVP, MEP, IOP, TAP, FVP, POP, VVP, ABP, and HEP all contributed to IL-6 production by macrophages in a significant dose-dependent manner, up 94.16% of the positive control. CMP, TAP, OSP, MEP, POP, VVP and ABP had the strongest effect at a concentration of 200 μg/mL, with IL-6 production of 60.30%, 4.37%, 71.48%, 9.85%, 4.06%, 47.92% and 5.82% of the positive control, respectively. FVP and HEP were most effective at a concentration of 100 μg/mL, and the production of IL-6 was 39.42% and 3.91% of the positive control group, respectively; IOP and CVP were most effective at a concentration of 50 μg/mL, and the production of IL-6 was 81.49% and 94.16% of the positive control group, respectively; PEP was not significantly different from the blank control group at all concentration gradients differences. Among them, OSP, IOP, CMP, and CVP were the most effective in promoting IL-6 production by macrophages all reaching 70%, while TAP, MEP, POP, ABP, HEP, and PEP were less effective in promoting IL-1β production by macrophages, less than 10%. (**Figures 9C, D**)

**Figure 10:**
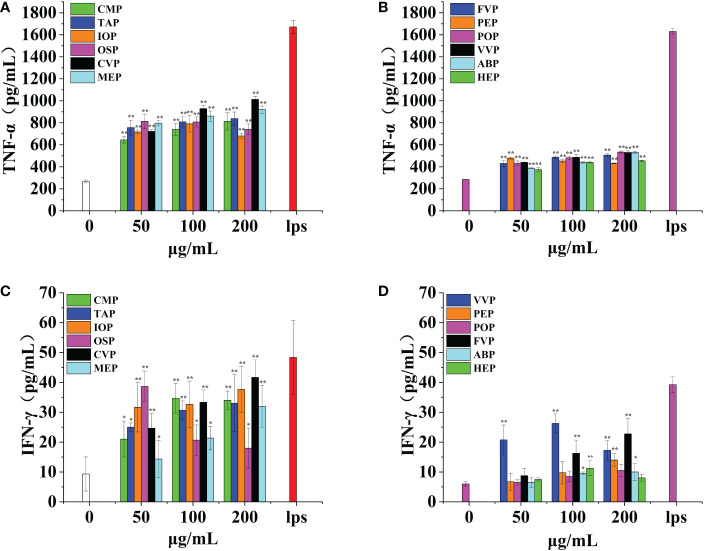
Effect of six rare edible mushrooms on TNF-α production in RAW 264.7 macrophages **(A)**. Effect of six common edible bacteria on TNF-α production in RAW 264.7 macrophages **(B)**, Effect of six rare edible bacteria on IFN-γ production in RAW 264.7 macrophages **(C)**, Effect of six common edible bacteria on IFN-γ production in RAW 264.7 macrophages **(D)**. *p < 0.05, **p < 0.01 indicates statistically significant difference compared to positive control group.

Gamma interferon plays a key role in host defense through antiviral, antiproliferative, and immunomodulatory functions. Gamma interferon effectively activates macrophages and directs B-cell immunoglobulin synthesis, type conversion, and secretion; gamma interferon plays an important role in several inflammatory disease processes such as autoimmunity and atherosclerosis.OSP, CMP, CVP, MEP, IOP, TAP, FVP, POP, VVP, ABP, HEP, and PEP all promote IFN-γ production by macrophages extremely significantly in a significant dose-dependent manner, reaching a minimum of 25.48% and a maximum of 106.06% of the positive control. The effects of TAP, IOP, CVP, MEP, FVP, PEP, POP, and ABP were strongest at a concentration of 200 μg/mL, when IFN-γ production was 92.12%, 83.03%, 106.06%, 80.00%, 57.96%, 35.67%, 26.75%, and 25.48% of the positive control group, respectively; CMP, VVP, and HEP showed a significant bellwether-type dose-dependent effect, with the strongest effect at a concentration of 100 μg/mL and IFN-γ production at 89.09%, 66.90%, and 28.66% of that of the positive control group, respectively.OSP had the strongest effect at a concentration of 50 μg/mL, and the amount of IFN-γ production was 95.15% of that in the positive control group. Among them, TAP, IOP, CVP, MEP, OSP, and CMP promoted IFN-γ production by macrophages with the best effect all reaching 70%, POP, ABP and HEP promoted IFN-γ production by macrophages with poorer effect below 30%. (**Figures 10A, B**)

Alpha-tumor necrosis factor (TNF-α) is a pro-inflammatory cytokine produced mainly by macrophages and monocytes and is involved in normal inflammatory and immune responses. Dysregulation of tumor necrosis factor-α production is thought to be associated with many human diseases, including Alzheimer’s disease, cancer, major depressive disorder, and enterocolitis.OSP, CMP, CVP, MEP, IOP, TAP, FVP, POP, VVP, ABP, HEP, and PEP all promote TNF-α production by macrophages extremely significantly and in a significant dose-dependent manner, reaching a minimum of 26.41% and a maximum of 60.58% of the positive control. The effects of CMP, TAP, CVP, MEP, FVP, POP, VVP, ABP, and HEP were strongest at a concentration of 200 μg/mL, when TNF-α production was 48.59%, 50.10%, 60.58%, 55.03%, 31.05%, 29.52%, 29.86%, 32.65% and 27.85% of the positive control group, respectively. The effect of IOP showed a significant bellwether-type dose dependence, with the strongest effect at a concentration of 100 μg/mL, and the TNF-α production was 47.26% of the positive control group; OSP and PEP showed the strongest effect at a concentration of 50 μg/mL, and the TNF-α production was 48.29% and 26.41% of the positive control group, respectively; Among them, CVP promoted TNF-α production by macrophages with the best effect reaching 60%, POP, VVP, PEP, and HEP promoted IFN-γ production by macrophages with poorer effect below 30%. all reached highly significant levels (p < 0.01). (**Figures 10C, D**).

### Comparison of the immune activity of twelve edible mushroom proteins

A comprehensive analysis of the effects of the above twelve edible mushroom proteins on the secretion of IL-1β, IL-6, IFN-γ, and TNF-α by mouse macrophages revealed that the proteins of different edible mushrooms showed different degrees of promoting the immune activity of macrophages. In this section, the immunomodulatory activities of twelve edible mushroom proteins were compared using the mathematical method of homogenization to calculate the modal lengths to screen the edible mushroom proteins with the best immune activity. The four original indices, IL-1β, IL-6, IFN-γ, and TNF-α, were first standardized and then homogenized to calculate ||X||2 using the mode length formula, and an ANOVA was performed on ||X||2 to reveal the differences in the immune activity of the twelve edible mushroom proteins. The immunoreactivity of the twelve edible mushroom proteins was compared as shown in the table, and the ||X||2 of the twelve proteins in the concentration range of 50-200 μg/mL was the largest for the TAP protein at 200 μg/mL, which was 1.31 ± 0.95. The ||X||2 of the six proteins at each concentration was subjected to ANOVA with them, and the results showed that there was a highly significant difference at each concentration (**P<0.01). In summary, among the six edible mushroom proteins, the immunomodulatory activities were TAP, CVP, MEP, OSP, IOP, CMP, VVP, FVP, PEP, POP, HEP, and ABP, in descending order of strength. (**Table 4**).

**Table 4:** Comparison of the immune activity of twelve edible mushroom proteins (n = 5).

	Concentration (μg/mL)	X_1_	X_2_	X_3_	X_4_	X | |^2^
**CMP**	50	0.13±0.03	0.37±0.05	0.64±0.37	0.39±0.02	0.17±0.01**
	100	0.25±0.07	0.49±0.06	1.07±0.62	0.44±0.04	0.05±0.02**
	200	0.30±0.04	0.60±0.10	0.95±0.44	0.49±0.05	0.09±0.07**
**TAP**	50	0.05±0.00	0.02±00	0.70±0.32	0.45±0.04	0.27±0.09**
	100	0.13±0.05	0.03±0.01	0.91±0.48	0.48±0.04	0.51±0.15**
	200	0.02±0.01	0.04±0.01	1.17±0.86	0.50±0.05	**1.31±0.95**
**IOP**	50	0.57±0.00	0.82±0.03	0.73±0.18	0.43±0.02	0.15±0.02**
	100 200	0.68±0.10	0.64±0.07	0.74±0.15	0.47±0.04	0.13±0.03**
		0.72±0.18	0.63±0.07	0.88±0.21	0.41±0.03	0.19±0.07**
**OSP**	50	0.87±0.00	0.64±0.04	1.11±0.55	0.49±0.04	0.40±0.12**
	100	1.00±0.15	0.65±0.02	0.68±0.45	0.48±0.02	0.27±0.13**
	200	0.97±0.03	0.72±0.07	0.45±0.20	0.44±0.04	0.20±0.03**
**CVP**	50	0.45±0.00	0.94±0.03	0.83±0.56	0.43±0.02	0.34±0.10**
	100	0.65±0.06	0.80±0.05	1.02±0.57	0.56±0.02	0.36±0.10**
	200	0.71±0.04	0.62±0.03	1.27±0.71	0.61±0.02	0.61±0.31**
**MEP**	50	0.08±0.00	0.01±0.00	0.57±0.48	0.48±0.03	0.32±0.12**
	100	0.17±0.08	0.02±0.00	0.55±0.19	0.52±0.03	0.13±0.08**
	200	0.11±0.04	0.10±0.02	0.94±0.51	0.55±0.04	0.53±0.34**
**FVP**	50	0.08±0.01	0.20±0.01	0.22±0.05	0.26±0.02	0.02±0.00**
	100	0.27±0.06	0.39±0.02	0.42±0.15	0.30±0.01	0.05±0.02**
	200	0.19±0.06	0.35±0.02	0.59±0.17	0.31±0.01	0.09±0.07**
**PEP**	50	0.15±0.04	0.01±0.00	0.17±0.07	0.29±0.01	0.03±0.00**
	100	0.27±0.02	0.01±0.00	0.24±0.08	0.28±0.01	0.03±0.00**
	200	0.30±0.09	0.00±0.00	0.36±0.05	0.26±0.01	0.04±0.01**
**POP**	50	0.08±0.03	0.01±0.00	0.17±0.04	0.27±0.01	0.02±0.00**
	100	0.32±0.06	0.01±0.00	0.21±0.03	0.30±0.01	0.03±0.01**
	200	0.14±0.01	0.04±0.01	0.27±0.07	0.33±0.01	0.03±0.01**
**VVP**	50	0.02±0.01	0.02±0.01	0.53±0.11	0.27±0.01	0.13±0.05**
	100	0.12±0.07	0.04±0.00	0.34±0.09	0.30±0.01	0.17±0.06**
	200	0.53±0.04	0.48±0.03	0.44±0.10	0.33±0.01	0.06±0.01**
**ABP**	50	0.02±0.01	0.01±0.00	0.17±0.04	0.24±0.00	0.02±0.00**
	100	0.11±0.01	0.05±0.00	0.24±0.03	0.27±0.01	0.02±0.00**
	200	0.22±0.04	0.06±0.00	0.26±0.10	0.33±0.01	0.03±0.01**
**HEP**	50	0.02±0.01	0.01±0.00	0.19±0.01	0.23±0.01	0.02±0.00**
	100	0.07±0.01	0.04±0.01	0.29±0.07	0.27±0.01	0.03±0.01**
	200	0.09±0.03	0.02±0.01	0.2±0.02	0.28±0.01	0.02±0.00**

The present data were expressed the mean ± SD.

^**^means significant difference between 200 μg/mL of TAP group, p < 0.01.

## Discussion

Edible mushrooms are considered to be a rich source of bioactive components (46, 47). Although polysaccharides in mushrooms have been frequently studied, only a few studies have focused on bioactive proteins in mushrooms. Several bioactive proteins have been recently purified from edible mushrooms. A protein (LEP91-3A2) was isolated from the liquid mycelial culture supernatant of Shiitake mushroom, which has antitumor effects on a variety of cancer cells (47). A family of fungal proteins (FIP-vvo, FIP-gts, FIP-fve, etc.) were identified in Straw mushroom, *G. lucidum*, and Golden mushroom and has been shown to have antitumor and immunomodulatory properties (3, 48, 49). However, so far, there is no comprehensive study on the immunomodulatory activity of common and precious edible mushrooms, leading us to be unclear about the strength of protein immunomodulatory ability of different edible mushrooms or the difference between common and precious edible mushrooms.

In the present study, we successfully extracted proteins from each of the twenty-two edible mushrooms, and to determine the potential biological activity of these proteins, we performed a series of experiments to determine their immunomodulatory potential and to evaluate the immunomodulatory activity of the extracted proteins systematically. Since macrophages are extremely functionally rich cells, they play a key role in host defense against bacterial infections due to their phagocytic, cytotoxic, and intracellular killing capabilities (50, 51).

However, in agreement with the literature, our results show that OSP, CMP, AAP, CVP, PIP, GLP, GFP, MEP, IOP, BP, TMSP, RAP, TAP, TMP, PEP, FVP, and VVP promote phagocytosis of neutrophils by macrophages and significantly enhance phagocytosis by RAW 264.7 macrophages, indicating that these edible mushroom proteins can enhance the immunomodulatory ability of mice. Upon stimulation with different concentrations of edible mushroom proteins, macrophages increased the production of NO, ROS, and pro-inflammatory cytokines, including IFN-γ, TNF-α, IL-6, and IL-1β. IL-1β is one of the effector molecules produced by macrophages, which can be involved in fever by inducing an acute phase protein response. It is a potent pro-inflammatory cytokine expressed on cells when there is viral infection or after inflammation has occurred. IL-1β expression is enhanced by increased expression of pattern recognition receptors (PRR) and Toll-like receptors (TLR) (52). Proteins such as OSP and CMP can enter macrophages to produce IL-1β, with OSP promoting IL-1β production by macrophages up to 99.70% of positive controls.Interleukin-6 (IL-6) is a multipotent cytokine that not only promotes the growth and differentiation of primary bone marrow-derived cells but also participates in the body’s immune defense, etc. CMP, IOP, and other proteins can enter macrophages to produce IL-6, with CVP promoting IL-6 production by macrophages up to 94.16% of the positive control. Studies have pointed out that the increase in IL-6 may be due to the activation of membrane receptors on the cell surface, which regulate the secretion and expression of the corresponding cytokines through the TLRs-mediated MAPK pathway (9, 16). Gamma interferons play a key role in host defense through antiviral, antiproliferative, and immunomodulatory functions. In several cell types, γ interferon induces cytokine production and upregulates the expression of different membrane proteins, which can effectively activate macrophages and direct the synthesis, type conversion, and secretion of B-cell immunoglobulins. proteins such as TAP and IOP can enter macrophages to produce IFN-γ, with TAP promoting IFN-γ production in macrophages up to 92.12% of the positive control. Tumor necrosis factor (TNF) is a group of cytokines secreted by activated mononuclear macrophages, NK cells, and T lymphocytes. Among them, TNF-α is the main immunomodulatory factor, which can initiate not only immune regulation and promote the activity of immune cells but also has various important biological effects such as anti-malignancy, anti-virus, coagulation, hematopoiesis, etc. It triggers the expression of vascular endothelial cells: it enhances leukocyte expression of adhesion molecules, promotes immune cell infiltration, and is able to enhance lymphocyte infiltration to the site of infection, which plays a crucial role in the early response to antiviral infection (53, 54).TAP, MEP, and other proteins can enter macrophages to produce TNF-α, reaching 50.10% and 55.03% of the positive control group, respectively. The highest rate of TNF-α production was 60.58% of the positive control. Studies have shown that purification from Chrysalis ferment significantly increased TNF-α production in macrophages and was associated with the TLR2/TLR4/Dectin-1 mediated NF-кB signaling pathway on the cell surface (55). A comprehensive analysis of the effects of the above twenty-two edible mushroom proteins on macrophage secretion of ROS, synthesis of NO, and phagocytosis of neutral red can reveal that different edible mushroom proteins present different degrees of promotion of macrophage immune activity, and overall, the immunomodulatory activity of precious edible mushrooms is stronger than that of common edible mushrooms, mainly in the best effect of precious edible mushroom proteins than common edible mushroom proteins. Further, the immunomodulatory activity of five edible mushroom proteins was compared using the mathematical method of homogenization to calculate the modal length to screen the edible mushroom proteins with the best immune activity.

The three raw indices of ROS secretion, NO, and phagocytosis were first normalized and then homogenized using the modal length formula to calculate ||X||^2^. The immunoreactivity of twenty-two edible bacteria proteins was compared as shown in **Table 3**. The top six valuable edible bacterial proteins with the highest ||X||^2^ in the concentration range of 25-200 μg/mL were CMP, CVP, IOP, TAP, MEP, and OSP||X||^2^ of 9.98 ± 1.31, 9.74 ± 1.16, 6.94 ± 1.45, 5.04 ± 0.35, 2.89 ± 0.46, and 2.74 ± 0.29, respectively. The first six common edible mushroom proteins VVP, PEP, ABP, FVP, POP, and HEP ||X|||^2^ were 1.65 ± 0.31, 1.4 ± 0.17, 1.34 ± 0.05, 1.25 ± 0.2, 1.15 ± 0.04 and 0.97 ± 0.08, respectively. The results showed that among the twenty-two edible mushroom proteins, the precious edible mushroom proteins were more common edible mushrooms and had higher immune activity. It was shown that medicinal fungi have a stronger immunomodulatory ability, and since CMP, CVP, IOP, TAP, MEP, and OSP are all valuable edible fungi, the reason for the stronger immunomodulatory effect of precious edible fungal proteins may be that medicinal fungi have some special proteins that are not found in common edible fungi (56).

Further studies on the screening of six precious edible mushrooms and six common edible mushroom proteins on macrophage secretion of IFN-γ, TNF-α, IL-6, and IL-1β showed that the precious edible mushroom proteins were more immunoreactive than the common edible mushroom proteins, with the strongest immunomodulation being TAP followed by CVP and MEP. Our results showed that edible mushroom proteins effectively enhanced macrophage phagocytosis and promoted the overproduction of ROS, NO, IFN-γ, TNF-α, IL-6, and IL-1β in macrophages, indicating that edible mushroom proteins could activate membrane receptors on the cell surface, regulate the production of different cytokines and promote the polarization of macrophages into classical M1-type macrophages, in which precious edible mushroom The immunomodulatory capacity was generally stronger than that of common edible mushrooms, further demonstrating that edible mushroom proteins can enhance immunity and have the potential to act as immunomodulators. According to our results, due to the immunomodulatory effects of different edible mushrooms, they may be good candidates for future immunomodulators, especially precious edible mushrooms that have great potential as immunomodulators.

## Conclusion

In this study, we extracted proteins from thirteen rare edible mushrooms and nine common edible mushrooms, respectively, and determined their effects on mouse macrophage immunity. We found that these different edible mushroom proteins significantly increased the proliferation, phagocytosis, ROS secretion, nitric oxide, and pro-inflammatory cytokines in mouse macrophages, exhibiting strong immunomodulatory effects. Compared with common edible mushroom proteins such as VVP and PEP, the precious edible mushroom proteins TAP, CVP, and other precious edible mushrooms showed better immunomodulatory effects after treatment, with TAP showing the strongest immune activity, CVP second only to TAP, MEP, and OSP having average immune effects, and IOP and CMP having poor immune effects. Our results suggest that edible mushroom proteins have excellent immunomodulatory effects and have great potential in immunomodulation, and valuable edible mushroom proteins may be good candidates for future immunomodulators to maximize their beneficial effects.

## Data availability statement

The original contributions presented in the study are included in the article/**Supplementary Material**. Further inquiries can be directed to the corresponding author.

## Author contributions

GM conceived and designed the research. JX and DX performed the experiments and analyzed the data. DX, NM, FP, AS, and QH wrote and edited the manuscript. All authors contributed to the article and approved the submitted version.
