# Supplementary material for: Immune regulatory functions of biologically active proteins from edible fungi
Source: Front Immunol. 2023 Jan 12;13:1034545. doi: 10.3389/fimmu.2022.1034545 (PMC9878603; doi:10.3389/fimmu.2022.1034545)
Supplement: Supplementary file 1 [file Table_1.docx]

**Table 1.** **Protein content of different edible mushrooms** **(n=6)**.

| **Edible mushrooms** | **Protein（%）** |
| --- | --- |
| ***Russula alutacea*** | 41.21±0.56 |
| ***Volvariella volvacea*** | 38.65±1.14 |
| ***Agrocybe aegerita*** | 38.65±0.13 |
| ***Morchella esculenta*** | 36.79±0.42 |
| ***Boletus*** | 35.52±0.25 |
| ***Tuber melanosporum*** | 29.60±0.08 |
| ***Agaricus bisporus*** | 29.23±0.21 |
| ***Pleurotus eryngii*** | 28.48±0.47 |
| ***Ganoderma lucidum*** | 27.48±0.03 |
| ***Ophiocordyceps sinensis*** | 26.31±0.05 |
| ***Cordyceps militaris*** | 25.65±0.49 |
| ***Lentinula edodes*** | 25.48±0.31 |
| ***Hypsizygus marmoreus*** | 24.67±0.21 |
| ***Flammulina velutipes*** | 24.40±0.31 |
| ***Phellinus igniarius*** | 24.02±0.16 |
| ***Pleurotus ostreatus*** | 22.67±0.28 |
| ***Termitornyces albuminosus*** | 21.52±0.16 |
| ***Inonotus obliquus*** | 20.44±0.18 |
| ***Hericium erinaceus*** | 20.23±0.06 |
| ***Grifola frondosa*** | 18.44±0.18 |
| ***Tricholoma matsutake Singer*** | 18.40±0.26 |
| ***Coriolus versicolor*** | 17.42±0.11 |

The present data were expressed the mean ± SD.

**Table 2.** **Composition of proteins (n=6)**.

|  | Protein | Polysaccharide | Cellulose | Mositure | Ash | uronic acid | Lignin | Unknown |
| --- | --- | --- | --- | --- | --- | --- | --- | --- |
| **CMP** | 75.82±0.10 | 8.61±0.06 | 6.47±0.1 | 1.91±0.11 | 2.69±0.19 | 1.13±0.11 | 1.81±0.01 | 4.13±0.35 |
| **PEP** | 73.35±0.14 | 5.72±0.35 | 8.87±0.11 | 2.28±0.06 | 5.71±0.18 | 1.00±0.15 | 0.83±0.06 | 2.24±0.39 |
| **POP** | 73.05±0.10 | 5.75±0.18 | 9.55±0.33 | 2.34±0.06 | 3.35±0.10 | 1.04±0.03 | 0.51±0.04 | 4.41±0.26 |
| **TAP** | 69.97±1.26 | 8.74±0.20 | 6.45±0.28 | 1.56±0.02 | 5.79±0.24 | 1.23±0.16 | 1.16±0.07 | 5.11±0.99 |
| **LEP** | 69.39±0.30 | 5.86±0.22 | 10.13±0.39 | 2.09±0.08 | 3.64±0.33 | 1.42±0.04 | 1.91±0.05 | 5.56±0.89 |
| **ABP** | 69.23±3.01 | 4.42±0.16 | 6.01±0.20 | 1.75±0.05 | 6.69±0.19 | 1.62±0.07 | 1.74±0.09 | 8.53±3.22 |
| **VVP** | 69.22±0.23 | 9.69±0.24 | 7.89±0.08 | 1.61±0.03 | 5.45±0.30 | 0.86±0.04 | 0.41±0.03 | 4.86±0.52 |
| **HEP** | 68.78±0.12 | 7.38±0.16 | 7.92±0.35 | 1.54±0.12 | 5.39±0.20 | 0.93±0.07 | 0.46±0.05 | 7.60±0.81 |
| **HMP** | 66.79±0.10 | 5.14±1.00 | 5.79±0.19 | 2.34±0.23 | 6.82±0.13 | 1.31±0.30 | 1.96±0.02 | 9.85±1.51 |
| **CVP** | 64.30±0.37 | 8.82±0.17 | 9.50±0.31 | 1.19±0.03 | 3.34±0.16 | 1.07±0.16 | 1.95±0.04 | 9.82±0.58 |
| **OSP** | 63.82±0.71 | 6.78±0.13 | 9.73±0.36 | 2.16±0.04 | 5.37±0.19 | 1.27±0.06 | 0.48±0.03 | 10.39±0.73 |
| **PIP** | 62.37±0.14 | 9.67±0.31 | 8.82±0.31 | 2.37±0.08 | 4.62±0.31 | 1.09±0.06 | 1.04±0.05 | 10.01±0.60 |
| **GLP** | 62.35±0.88 | 9.08±0.42 | 9.19±0.34 | 1.37±0.06 | 6.52±0.20 | 0.81±0.06 | 1.87±0.03 | 8.80±0.55 |
| **MEP** | 62.21±0.48 | 9.50±0.35 | 10.04±0.49 | 1.16±0.02 | 5.72±0.14 | 0.82±0.03 | 0.76±0.06 | 10.04±1.07 |
| **RAP** | 62.02±0.37 | 8.74±0.31 | 10.69±0.17 | 1.15±0.01 | 5.56±0.22 | 0.79±0.02 | 0.48±0.08 | 10.56±0.44 |
| **IOP** | 61.80±0.31 | 8.94±0.51 | 9.26±0.36 | 1.53±0.02 | 6.62±0.17 | 1.05±0.04 | 1.01±0.84 | 9.78±0.92 |
| **BP** | 61.16±0.32 | 9.22±0.18 | 9.44±0.61 | 3.01±0.14 | 5.76±0.38 | 0.88±0.02 | 1.21±0.04 | 9.31±0.88 |
| **AAP** | 60.92±0.47 | 8.74±0.18 | 10.16±0.39 | 2.16±0.22 | 3.59±0.15 | 1.25±0.07 | 1.72±0.01 | 11.45±0.83 |
| **TMP** | 60.53±0.84 | 6.82±0.36 | 9.97±0.26 | 2.46±0.07 | 7.57±0.21 | 1.14±0.03 | 0.50±0.07 | 11.00±0.79 |
| **FVP** | 60.40±0.86 | 8.10±0.13 | 11.04±0.43 | 3.40±0.44 | 6.40±0.27 | 1.01±0.14 | 1.44±0.03 | 8.20±1.27 |
| **TMSP** | 60.30±0.84 | 8.76±0.24 | 10.68±0.28 | 3.65±0.45 | 7.53±0.39 | 0.87±0.03 | 1.26±0.03 | 6.94±0.16 |
| **GFP** | 60.06±2.16 | 8.86±0.44 | 7.66±0.29 | 3.07±0.21 | 7.69±0.09 | 1.03±0.09 | 2.14±0.03 | 9.49±1.66 |

The present data were expressed the mean ± SD.

**Table 3. Comparison of the immune activity of twenty-two edible mushroom proteins (n=5).**

| Groups | Concentration(μg/mL) | X_1_ | X_2_ | X_3_ | \|\|X\|\|^2^ |
| --- | --- | --- | --- | --- | --- |
| **OSP** | 25 | 2.11±0.14 | 3.17±0.26 | 1.86±0.11 | 2.66±0.45** |
|  | 50  100  200 | 1.74±0.13 | 2.94±0.19 | 2.93±0.12 | 2.74±0.29** |
|  |  | 1.73±0.10 | 2.59±0.16 | 3.03±0.21 | 2.60±0.31** |
|  |  | 1.81±0.11 | 2.39±0.18 | 2.98±0.20 | 2.42±0.30** |
| **CMP** | 25  50  100  200 | 1.33±0.10 | 3.97±0.06 | 1.33±0.12 | 5.82±0.28** |
|  |  | 1.46±0.15 | 4.29±0.13 | 1.50±0.14 | 6.67±0.74** |
|  |  | 1.97±0.21 | 4.40±0.07 | 1.56±0.18 | 6.33±0.44** |
|  |  | 2.22±0.12 | 5.43±0.28 | 1.90±0.05 | **9.98±1.31** |
| **AAP** | 25  50  100  200 | 1.47±0.09 | 1.13±0.09 | 1.26±0.04 | 0.61±0.04** |
|  |  | 1.64±0.08 | 1.23±0.11 | 1.78±0.21 | 0.94±0.16** |
|  |  | 1.61±0.14 | 1.46±0.10 | 1.17±0.28 | 0.79±0.08** |
|  |  | 1.95±0.15 | 1.63±0.17 | 1.47±0.04 | 1.05±0.14** |
| **CVP** | 25  50  100  200 | 1.18±0.06 | 1.13±0.12 | 1.91±0.23 | 1.00±0.29** |
|  |  | 1.57±0.13 | 1.29±0.14 | 3.45±0.13 | 3.82±0.38** |
|  |  | 1.11±0.08 | 1.90±0.32 | 4.35±0.21 | 7.08±1.39** |
|  |  | 1.16±0.05 | 2.83±0.15 | 5.25±0.22 | 9.74±1.16 |
| **PIP** | 25  50  100  200 | 2.15±0.11 | 2.34±0.12 | 1.34±0.05 | 1.62±0.19** |
|  |  | 1.96±0.26 | 2.18±0.12 | 1.32±0.03 | 1.41±0.13** |
|  |  | 1.89±0.21 | 2.13±0.14 | 2.18±0.17 | 1.50±0.15** |
|  |  | 1.85±0.15 | 1.96±0.11 | 2.24±0.08 | 1.43±0.09** |
| **GLP** | 25  50  100  200 | 1.50±0.11 | 2.34±0.12 | 2.00±0.11 | 2.21±0.08** |
|  |  | 1.94±0.10 | 2.18±0.12 | 2.79±0.26 | 2.20±0.28** |
|  |  | 1.18±0.04 | 2.13±0.14 | 2.65±0.23 | 2.06±0.35** |
|  |  | 1.01±0.18 | 1.96±0.11 | 1.56±0.13 | 1.06±0.37** |
| **GFP** | 25  50  100  200 | 1.14±0.04 | 0.99±0.08 | 1.65±0.28 | 0.77±0.28** |
|  |  | 1.37±0.17 | 1.01±0.09 | 2.66±0.13 | 2.18±0.32** |
|  |  | 1.25±0.06 | 1.12±0.08 | 2.41±0.17 | 1.68±0.27** |
|  |  | 0.92±0.11 | 1.44±0.09 | 1.56±0.27 | 0.77±0.21** |
| **MEP** | 25  50  100  200 | 0.89±0.07 | 1.10±0.10 | 1.86±0.11 | 0.96±0.12** |
|  |  | 1.83±0.12 | 1.04±0.09 | 2.93±0.12 | 2.57±0.31** |
|  |  | 1.31±0.05 | 1.29±0.23 | 3.03±0.21 | 2.89±0.46** |
|  |  | 1.34±0.12 | 1.69±0.17 | 2.98±0.20 | 2.54±0.38** |
| **IOP** | 25  50  100  200 | 0.88±0.09 | 2.81±0.10 | 1.56±0.14 | 2.52±0.21** |
|  |  | 0.85±0.06 | 3.52±0.13 | 1.81±0.11 | 4.32±0.39** |
|  |  | 0.82±0.07 | 3.86±0.12 | 1.66±0.08 | 5.76±0.52** |
|  |  | 0.81±0.12 | 4.21±0.33 | 1.90±0.07 | 6.94±1.45** |
| **BP** | 25  50  100  200 | 0.89±0.06 | 1.13±0.03 | 1.64±0.14 | 0.72±0.13** |
|  |  | 0.69±0.05 | 1.07±0.04 | 2.15±0.27 | 1.54±0.49** |
|  |  | 0.67±0.10 | 1.06±0.04 | 2.14±0.11 | 1.51±0.25** |
|  |  | 0.71±0.06 | 1.09±0.05 | 2.69±0.12 | 2.70±0.30** |
| **TMSP** | 25  50  100  200 | 1.73±0.07 | 1.10±0.13 | 1.93±0.13 | 1.10±0.13** |
|  |  | 1.68±0.11 | 1.16±0.06 | 1.82±0.11 | 0.98±0.07** |
|  |  | 1.46±0.09 | 1.14±0.06 | 1.57±0.23 | 0.74±0.16** |
|  |  | 1.41±0.22 | 1.22±0.08 | 1.83±0.21 | 0.93±0.19** |
| **RAP** | 25  50  100  200 | 1.21±0.06 | 1.35±0.14 | 1.62±0.06 | 0.72±0.05** |
|  |  | 1.79±0.11 | 1.19±0.15 | 2.05±0.10 | 1.21±0.17** |
|  |  | 2.42±0.10 | 1.11±0.12 | 2.17±0.11 | 1.80±0.14** |
|  |  | 2.67±0.13 | 1.04±0.14 | 2.55±0.12 | 2.43±0.28** |
| **TAP** | 25  50  100  200 | 1.51±0.08 | 1.94±0.18 | 1.81±0.15 | 1.12±0.15** |
|  |  | 1.56±0.08 | 2.83±0.16 | 1.94±0.12 | 2.12±0.23** |
|  |  | 2.09±0.11 | 3.56±0.26 | 2.24±0.11 | 3.34±0.52** |
|  |  | 2.72±0.18 | 4.4±0.16 | 2.70±0.22 | 5.04±0.35** |
| **TMP** | 25  50  100  200 | 1.51±0.1 | 1.24±0.13 | 1.64±0.06 | 0.77±0.07** |
|  |  | 1.77±0.17 | 1.27±0.12 | 1.58±0.12 | 0.89±0.13** |
|  |  | 1.91±0.11 | 1.31±0.05 | 2.16±0.19 | 1.35±0.19** |
|  |  | 2.56±0.24 | 1.47±0.04 | 2.29±0.17 | 1.92±0.36** |
| **PEP** | 25  50  100  200 | 0.80±0.05 | 1.12±0.08 | 1.69±0.19 | 0.81±0.23** |
|  |  | 0.97±0.12 | 1.13±0.03 | 1.98±0.18 | 1.11±0.28** |
|  |  | 1.01±0.06 | 1.21±0.06 | 2.22±0.11 | 1.40±0.17** |
|  |  | 1.03±0.12 | 1.44±0.16 | 1.54±0.18 | 0.71±0.15** |
| **FVP** | 25  50  100  200 | 0.90±0.11 | 0.99±0.08 | 2.05±0.12 | 1.25±0.20** |
|  |  | 0.98±0.07 | 1.03±0.14 | 1.41±0.17 | 0.53±0.13** |
|  |  | 0.84±0.03 | 1.05±0.14 | 1.40±0.13 | 0.54±0.08** |
|  |  | 0.80±0.10 | 1.12±0.11 | 1.38±0.18 | 0.55±0.12** |
| **VVP** | 25  50  100  200 | 0.99±0.07 | 2±0.1 | 1.47±0.15 | 1.10±0.11** |
|  |  | 0.93±0.08 | 2.06±0.14 | 2.18±0.34 | 1.65±0.31** |
|  |  | 0.95±0.12 | 2.04±0.17 | 1.86±0.15 | 1.32±0.23** |
|  |  | 1.03±0.11 | 1.79±0.08 | 1.96±0.10 | 1.16±0.06** |
| **HEP** | 25  50  100  200 | 1.02±0.15 | 1.02±0.05 | 1.21±0.03 | 0.42±0.02** |
|  |  | 0.98±0.08 | 1.06±0.08 | 1.29±0.09 | 0.45±0.05** |
|  |  | 1.01±0.13 | 1.15±0.15 | 1.63±0.06 | 0.71±0.05** |
|  |  | 1.11±0.07 | 1.24±0.16 | 1.91±0.08 | 0.97±0.08** |
| **POP** | 25  50  100  200 | 0.99±0.07 | 1.07±0.09 | 1.63±0.05 | 0.69±0.04** |
|  |  | 1.00±0.05 | 1.15±0.16 | 1.69±0.04 | 0.76±0.04** |
|  |  | 0.99±0.06 | 1.23±0.13 | 1.87±0.04 | 0.93±0.05** |
|  |  | 1.01±0.06 | 1.63±0.17 | 2.01±0.05 | 1.15±0.04** |
| **LEP** | 25  50  100  200 | 0.99±0.05 | 1.02±0.06 | 1.21±0.03 | 0.41±0.02** |
|  |  | 0.99±0.04 | 1.06±0.09 | 1.29±0.09 | 0.45±0.05** |
|  |  | 1.02±0.04 | 1.18±0.13 | 1.28±0.08 | 0.48±0.07** |
|  |  | 1.11±0.08 | 1.3±0.15 | 1.51±0.06 | 0.64±0.06** |
| **ABP** | 25  50  100  200 | 1.00±0.11 | 1.2±0.09 | 1.70±0.16 | 0.79±0.18** |
|  |  | 0.99±0.13 | 1.24±0.14 | 1.98±0.15 | 1.10±0.22** |
|  |  | 1.01±0.02 | 1.26±0.09 | 2.19±0.05 | 1.34±0.05** |
|  |  | 1.01±0.09 | 1.94±0.21 | 1.62±0.11 | 1.09±0.25** |
| **HMP** | 25  50  100  200 | 1.01±0.05 | 0.97±0.09 | 1.21±0.03 | 0.40±0.03** |
|  |  | 0.99±0.07 | 1.06±0.1 | 1.29±0.09 | 0.46±0.05** |
|  |  | 0.98±0.11 | 1.16±0.11 | 1.28±0.08 | 0.47±0.06** |
|  |  | 1.02±0.06 | 1.25±0.11 | 1.39±0.08 | 0.55±0.06** |

The present data were expressed the mean ± SD.

^**^ means significant difference between 200 μg/mL of CMP group, p < 0.01

**Table 4. Comparison of the immune activity of twelve edible mushroom proteins(n=5)**.

|  | Concentration(μg/mL) | | X_1_ | | X_2_ | | X_3_ | | X_4_ | | \| \| X \| \|^2^ | |
| --- | --- | --- | --- | --- | --- | --- | --- | --- | --- | --- | --- | --- |
| **CMP** | 50 | 0.13±0.03 | | 0.37±0.05 | | 0.64±0.37 | | 0.39±0.02 | | 0.17±0.01** | |  |
|  | 100 | 0.25±0.07 | | 0.49±0.06 | | 1.07±0.62 | | 0.44±0.04 | | 0.05±0.02** | |  |
|  | 200 | 0.30±0.04 | | 0.60±0.10 | | 0.95±0.44 | | 0.49±0.05 | | 0.09±0.07** | |  |
| **TAP** | 50 | 0.05±0.00 | | 0.02±00 | | 0.70±0.32 | | 0.45±0.04 | | 0.27±0.09** | |  |
|  | 100 | 0.13±0.05 | | 0.03±0.01 | | 0.91±0.48 | | 0.48±0.04 | | 0.51±0.15** | |  |
|  | 200 | 0.02±0.01 | | 0.04±0.01 | | 1.17±0.86 | | 0.50±0.05 | | **1.31±0.95** | |  |
| **IOP** | 50 | 0.57±0.00 | | 0.82±0.03 | | 0.73±0.18 | | 0.43±0.02 | | 0.15±0.02** | |  |
|  | 100  200 | 0.68±0.10 | | 0.64±0.07 | | 0.74±0.15 | | 0.47±0.04 | | 0.13±0.03** | |  |
|  |  | 0.72±0.18 | | 0.63±0.07 | | 0.88±0.21 | | 0.41±0.03 | | 0.19±0.07** | |  |
| **OSP** | 50 | 0.87±0.00 | | 0.64±0.04 | | 1.11±0.55 | | 0.49±0.04 | | 0.40±0.12** | |  |
|  | 100 | 1.00±0.15 | | 0.65±0.02 | | 0.68±0.45 | | 0.48±0.02 | | 0.27±0.13** | |  |
|  | 200 | 0.97±0.03 | | 0.72±0.07 | | 0.45±0.20 | | 0.44±0.04 | | 0.20±0.03** | |  |
| **CVP** | 50 | 0.45±0.00 | | 0.94±0.03 | | 0.83±0.56 | | 0.43±0.02 | | 0.34±0.10** | |  |
|  | 100 | 0.65±0.06 | | 0.80±0.05 | | 1.02±0.57 | | 0.56±0.02 | | 0.36±0.10** | |  |
|  | 200 | 0.71±0.04 | | 0.62±0.03 | | 1.27±0.71 | | 0.61±0.02 | | 0.61±0.31** | |  |
| **MEP** | 50 | 0.08±0.00 | | 0.01±0.00 | | 0.57±0.48 | | 0.48±0.03 | | 0.32±0.12** | |  |
|  | 100 | 0.17±0.08 | | 0.02±0.00 | | 0.55±0.19 | | 0.52±0.03 | | 0.13±0.08** | |  |
|  | 200 | 0.11±0.04 | | 0.10±0.02 | | 0.94±0.51 | | 0.55±0.04 | | 0.53±0.34** | |  |
| **FVP** | 50 | 0.08±0.01 | | 0.20±0.01 | | 0.22±0.05 | | 0.26±0.02 | | 0.02±0.00** | |  |
|  | 100 | 0.27±0.06 | | 0.39±0.02 | | 0.42±0.15 | | 0.30±0.01 | | 0.05±0.02** | |  |
|  | 200 | 0.19±0.06 | | 0.35±0.02 | | 0.59±0.17 | | 0.31±0.01 | | 0.09±0.07** | |  |
| **PEP** | 50 | 0.15±0.04 | | 0.01±0.00 | | 0.17±0.07 | | 0.29±0.01 | | 0.03±0.00** | |  |
|  | 100 | 0.27±0.02 | | 0.01±0.00 | | 0.24±0.08 | | 0.28±0.01 | | 0.03±0.00** | |  |
|  | 200 | 0.30±0.09 | | 0.00±0.00 | | 0.36±0.05 | | 0.26±0.01 | | 0.04±0.01** | |  |
| **POP** | 50 | 0.08±0.03 | | 0.01±0.00 | | 0.17±0.04 | | 0.27±0.01 | | 0.02±0.00** | |  |
|  | 100 | 0.32±0.06 | | 0.01±0.00 | | 0.21±0.03 | | 0.30±0.01 | | 0.03±0.01** | |  |
|  | 200 | 0.14±0.01 | | 0.04±0.01 | | 0.27±0.07 | | 0.33±0.01 | | 0.03±0.01** | |  |
| **VVP** | 50 | 0.02±0.01 | | 0.02±0.01 | | 0.53±0.11 | | 0.27±0.01 | | 0.13±0.05** | |  |
|  | 100 | 0.12±0.07 | | 0.04±0.00 | | 0.34±0.09 | | 0.30±0.01 | | 0.17±0.06** | |  |
|  | 200 | 0.53±0.04 | | 0.48±0.03 | | 0.44±0.10 | | 0.33±0.01 | | 0.06±0.01** | |  |
| **ABP** | 50 | 0.02±0.01 | | 0.01±0.00 | | 0.17±0.04 | | 0.24±0.00 | | 0.02±0.00** | |  |
|  | 100 | 0.11±0.01 | | 0.05±0.00 | | 0.24±0.03 | | 0.27±0.01 | | 0.02±0.00** | |  |
|  | 200 | 0.22±0.04 | | 0.06±0.00 | | 0.26±0.10 | | 0.33±0.01 | | 0.03±0.01** | |  |
| **HEP** | 50 | 0.02±0.01 | | 0.01±0.00 | | 0.19±0.01 | | 0.23±0.01 | | 0.02±0.00** | |  |
|  | 100 | 0.07±0.01 | | 0.04±0.01 | | 0.29±0.07 | | 0.27±0.01 | | 0.03±0.01** | |  |
|  | 200 | 0.09±0.03 | | 0.02±0.01 | | 0.2±0.02 | | 0.28±0.01 | | 0.02±0.00** | |  |

The present data were expressed the mean ± SD.

^**^ means significant difference between 200 μg/mL of TAP group, p < 0.01.
